# Boosting the photocatalytic H_2_O_2_ production of covalent organic frameworks with a heteroatom-locked acceptor and gas diffusion system

**DOI:** 10.1039/d5sc05346c

**Published:** 2025-11-08

**Authors:** Qianshuo Nan, Jing Ning, Bing Han, Hongtao Wei, Xuefeng Wang, Ying-Ying Gu, Shengxiang Zhou, Guangqiang Cao, Guangze Zhang, Xuehui Li, Yonggang Jia, Long Hao

**Affiliations:** a College of Chemistry and Chemical Engineering, China University of Petroleum (East China) Qingdao 266580 P. R. China yingyinggu@upc.edu.cn; b College of Chemistry and Pharmaceutical Sciences, Qingdao Agricultural University No. 700 Changcheng Road Qingdao 266109 P. R. China wxf@qau.edu.cnhaol@qau.edu.cn; c MOE Key Laboratory of Resources and Environmental Systems Optimization, College of Environmental Science and Engineering, North China Electric Power University Beijing 102206 P.R. China; d Marine Science Research Institute of Shandong Province 7 Youyun Road Qingdao 266104 China; e Shandong Provincial Key Laboratory of Marine Environment and Geological Engineering, Key Laboratory of Marine Environment and Ecology, Ocean University of China Qingdao 266100 China yonggang@ouc.edu.cn

## Abstract

Simultaneously improving charge carrier separation and surface reaction efficiency is crucial for enhancing the photocatalytic H_2_O_2_ production efficiency of covalent organic frameworks (COFs). Here, a heteroatom-lock strategy is introduced into the acceptor structure of COFs, with the “lock” effect to enhance the coplanarity and conjugation, and the “heteroatom” effect to improve the O_2_ adsorption. It turns out that the photocatalytic H_2_O_2_ production yield of the N-heteroatom locked COF (2.08 mmol g^−1^ h^−1^ under pure water and air conditions) is 2.1 times that of the S-heteroatom locked COF and 4.7 times that of the original COF. Experimental results and theoretical calculations reveal that the heteroatom-lock-induced H_2_O_2_ production enhancement of COFs is attributed to their lower exciton binding energy (*E*_b_) and smaller charge transfer resistance, together with the bigger O_2_ adsorption energy and lower transition state energy of the intermediates. Additionally, a novel gas diffusion reaction system is developed to further improve the O_2_ diffusion efficiency, which not only enhances the photocatalytic H_2_O_2_ production yield to 4.06 mmol g^−1^ h^−1^, but also realizes the immobilization and efficient recycling of the COF catalyst. This study provides new insights into the rational design of COF-based photocatalysts, and offers a novel approach for the reaction system of photocatalytic H_2_O_2_ production.

## Abstract 2

Simultaneously improving charge carrier separation and surface reaction efficiency is crucial for enhancing the photocatalytic H_2_O_2_ production efficiency of covalent organic frameworks (COFs).

## Graphical abstract

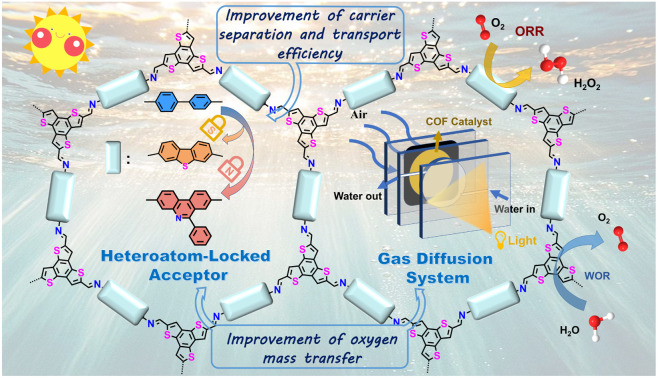

## Introduction

Given the worsening global energy crisis and environmental degradation, the need for sustainable and non-polluting energy solutions is becoming increasingly urgent. Hydrogen peroxide (H_2_O_2_) is a carbon-neutral fuel alternative with an energy density comparable to that of hydrogen,^1–3^ and its crucial role in sterilization, bleaching, organic synthesis, and environmental remediation with harmless by-product makes it a versatile environmentally friendly oxidant.^4,5^ Currently, industrial H_2_O_2_ production relies on the anthraquinone (AQ) process, which faces inherent drawbacks such as high energy consumption from multi-stage hydrogenation/oxidation cycles and pollution from organic by-products.^6,7^ In comparison, the solar-driven H_2_O_2_ production route based on semiconductor photocatalysts with atmospheric O_2_ and H_2_O as raw materials, exhibits higher atomic economy (theoretical yield of 100%) and milder reaction conditions (ambient temperature and pressure), which consequently has become a research frontier in sustainable chemistry.^8–10^ However, the current efficiency of photocatalytic H_2_O_2_ production remains low, necessitating the rational design and development of high-performance photocatalysts and catalytic reaction systems.

Previous studies have shown that traditional inorganic photocatalysts, such as TiO_2_,^11–13^ BiVO_4_,^14,15^ and metal sulfides,^16,17^ exhibit limited performance in photocatalytic H_2_O_2_ production. In contrast, organic polymeric photocatalysts, including graphitic carbon nitride (C_3_N_4_), conjugated organic polymers (COPs), and covalent organic frameworks (COFs) display significantly superior catalytic activity and stability.^18–20^ Notably, COF-based catalysts have received extensive attention since their first reported application in photocatalytic H_2_O_2_ production in 2020.^21^ This is not only attributed to the high specific surface area and ordered through-pore structure of COFs, which provide a large number of accessible active sites, but also to their molecular-level controllable framework structures and linkage types, which enable precise modulation of the catalyst's photoresponse, as well as the thermodynamics and kinetics of the reaction processes.^22–24^

At present, the photocatalytic H_2_O_2_ production efficiency of COFs still presents substantial room for improvement, which essentially needs in-depth investigation of the relationship between the COF structure and the fundamental photocatalytic processes (photoabsorption, carrier separation/transport, and surface redox reactions).^25^ Normally, photoabsorption can be properly tuned by the monomer and linkage types of the COF.^26–31^ However, the carrier separation/transport is more complicated, which is not only decided by the skeleton structure of the COF,^32–37^ but also affected by its microstructures, including conjugation,^38–40^ heteroatom-containing species (*e.g.* S/O/Se/N),^41,42^ position/content of nitrogen,^43–45^ or surface functional groups^46–49^ in the COF monomers. As for the surface redox reactions, the mass transport requires greater attention,^50–52^ especially for the O_2_-related species, in which heteroatoms with high oxygen affinity (*e.g.* N/S) are usually introduced,^53–56^ and a flow reaction system has also been developed recently.^57^ Therefore, to improve the overall efficiency of COF catalysts, it is necessary to consider the synergistic effect of the monomer structure on different photocatalytic processes. Meanwhile, developing a more reasonable reaction system may also be an effective approach.

**Scheme 1:**
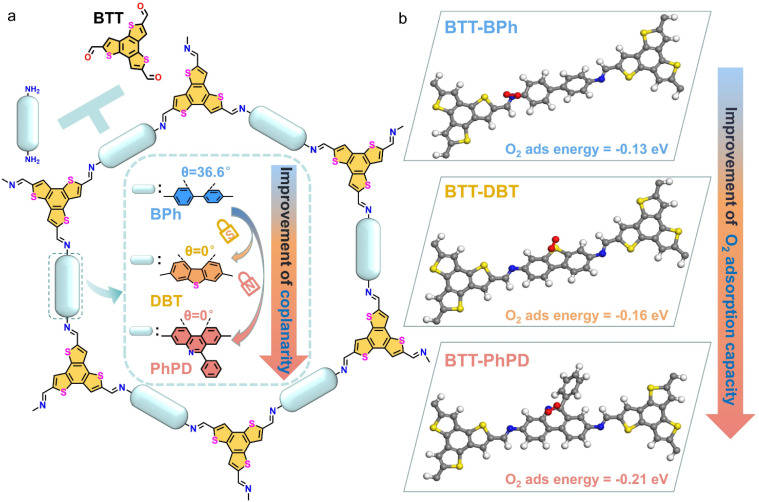
(a) Schematic representation for the construction of COFs: BTT-BPh, BTT-DBT and BTT-PhPD with the inset showing the heteroatom-lock strategy with improved coplanarity; (b) simulated schematic of O_2_ adsorption on the COF surface and the corresponding O_2_ adsorption energy (atom colour: N, blue; S, yellow; O, red; C, grey; H, white).

Herein, a heteroatom-lock strategy is applied in the acceptor part of the COF (Scheme 1a), specifically, S (for 3,7-diaminodibenzothiophene, DBT) or N (for 6-phenylphenanthridine-3,8-diamine, PhPD) atom is used to lock the rotation of the diaminobiphenyl (BPh) acceptor (the corresponding donor part of the COFs is benzotrithiophenetricarbaldehyde, BTT, a widely-used donor developed by our group^58–61^), in which the improvement of coplanarity (“lock” effect) aims to enhance the charge carrier separation/transport; meanwhile, the introduction of the S/N heteroatom is to improve the O_2_ adsorption capacity (see the simulated O_2_ adsorption energy of the structural units of COFs in Scheme 1b: −0.13 eV for BTT-BPh, −0.16 eV for S-locked BTT-DBT, and −0.21 eV for N-locked BTT-PhPD). It turns out that the photocatalytic H_2_O_2_ production yield of the N-locked COF (2.08 mmol g^−1^ h^−1^ under pure water and air conditions) is 2.1 times that of the S-locked COF and 4.7 times that of the original COF. Experimental results and theoretical calculations reveal that the heteroatom-lock-induced H_2_O_2_ production enhancement of the COF is attributed to its lower exciton binding energy (*E*_b_) and smaller charge transfer resistance, together with the bigger O_2_ adsorption energy and lower transition state energy of the intermediates. Additionally, a gas diffusion system (a simplified system similar to the O_2_-involved heterogeneous electrocatalytic system^62^) is also introduced to improve the O_2_ diffusion efficiency, which not only further enhances the photocatalytic H_2_O_2_ yield to 4.06 mmol g^−1^ h^−1^, but also realizes the immobilization and efficient recycling of the COF catalyst. This work elucidates the synergistic effect of the heteroatom-locked acceptor and the gas diffusion reaction system toward improvement of the fundamental photocatalytic processes, providing valuable insights for the rational design of efficient COF-based photocatalysts and reaction systems.

## Results and discussion

**Fig. 1:**
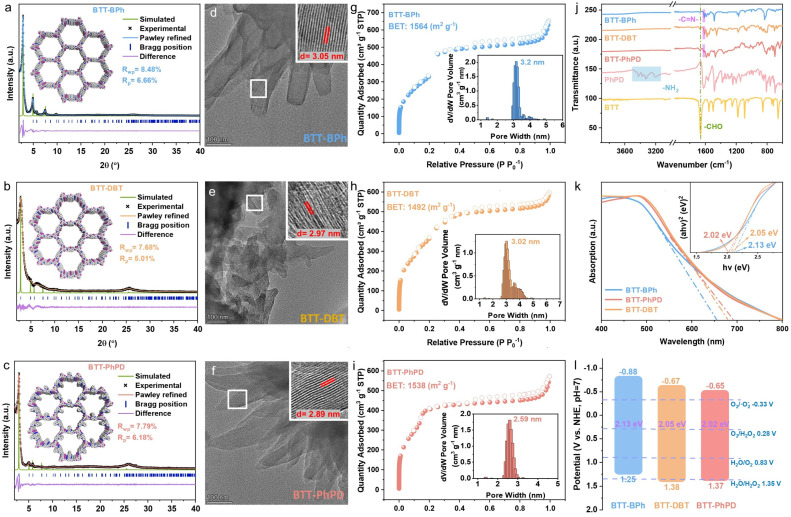
(a–c) Experimental and simulated XRD patterns with insets showing the packing models; (d–f) typical TEM images of the COFs with insets showing enlarged images of the indicated areas; (g–i) nitrogen adsorption–desorption isotherms with insets showing the pore size distribution profiles; (j) FT-IR spectra compared with the monomers PhPD and BTT; (k) ultraviolet (UV)/vis DRS spectra with the inset showing the Tauc plots; and (l) energy level diagrams of the COFs.

The COFs were constructed by the classic solvothermal method with BTT (synthesized based on our previous report^58,59^) as the donor part, and BPh (original)/DBT (S-locked)/PhPD (N-locked) as the acceptor part (Scheme 1a, details in the SI). The crystal structures of the corresponding COFs were elucidated by powder X-ray diffraction (PXRD) in comparison with the theoretical simulations (Fig. 1a–c): the experimental diffraction patterns of the COFs all align well with the calculated results derived from Forcite geometric simulations and Pawley refinement (see their fractional atomic coordinates in Tables S1–S3), demonstrating their AA-eclipsed stacking structures. All XRD patterns exhibit prominent diffraction peaks in low-angle regions, corresponding to the (100) planes of the primitive hexagonal lattice. The narrow full width at half maximum (FWHM) of the (100) plane peaks in the COFs indicates that they all possess good crystallinity. The clear lattice fringes observed in transmission electron microscope (TEM) images of the COFs provided additional confirmation of the periodic framework structures with high crystallinity (Fig. 1d–f, and S1).

Surface area and porosity of the COFs were evaluated through N_2_ adsorption–desorption isotherms at 77 K (Fig. 1g–i). All the COFs demonstrate similar and large Brunauer–Emmett–Teller (BET) surface areas: 1564 m^2^ g^−1^ for BTT-BPh, 1492 m^2^ g^−1^ for BTT-DBT, and 1538 m^2^ g^−1^ for BTT-PhPD (Fig. S2), and the corresponding average pore sizes are 3.2 nm (BTT-BPh), 3.02 nm (BTT-DBT), and 2.59 nm (BTT-PhPD), respectively (insets in Fig. 1g–i). The high surface areas and abundant mesopores provide numerous active sites for the photocatalytic reactions, while the similar surface areas enable a direct comparison of the structure–function relationship among the three COFs. FT-IR spectra of the COFs in Fig. 1j and S3 exhibit the reduction of amino group (–NH_2_) stretching vibration peaks at 3200–3400 cm^−1^ and carbonyl group (–C=O) stretching vibration peaks at ∼1660 cm^−1^ compared to the monomers, while displaying the appearance of imine (–C=N–) stretching vibration peaks at 1605–1617 cm^−1^, which confirms the formation of imine bonds through the condensation of the aldehyde and amine monomers. Besides, the thermogravimetric analysis (TGA) data demonstrate that all the COFs exhibit no obvious weight-loss before 230 °C, displaying their good thermal stability (Fig. S4). Water contact angle measurements for the three COFs indicate that the incorporation of heteroatoms leads to a moderate enhancement in the overall hydrophilicity of the materials (Fig. S5).

The light-harvesting properties of the COFs were analysed using UV-vis diffuse reflectance spectroscopy (UV-vis DRS) (Fig. 1k). The COFs all exhibit strong light absorption in the visible-light region (digital photos in Fig. S6), and with the heteroatom-lock, the light absorption edges of BTT-DBT and BTT-PhPD all red-shift. Correspondingly, the bandgaps of BTT-PhPD (2.02 eV) and BTT-DBT (2.05 eV) are smaller than that of BTT-BPh (2.13 eV) (see Tauc plots as the inset in Fig. 1k). Comparative analysis of the N 1s XPS spectra for the three COFs (Fig. S7) reveals that the imine N binding energies in both the N-locked BTT-PhPD (398.61 eV) and S-locked BTT-DBT (398.52 eV) are lower than that of pristine BTT-BPh (398.72 eV), which indicates a higher electron density around the N atoms. The enhanced electron density at the imine N can be rationally explained by the extended conjugation within the heteroatom-locked skeleton, which facilitates more efficient electron delocalization onto the N atoms. The red-shifted absorption edges and the decreased imine N binding energies indirectly suggest an extension of the π-conjugation. The MS curves of the COFs all exhibit a positive slope, indicating their n-type semiconductor nature (Fig. S8). The MS curves also indicate that the conduction band minimum (CBM) potentials of BTT-BPh, BTT-DBT, and BTT-PhPD are −0.88 V, −0.67 V and −0.65 V, respectively. According to the equation *E*_g_ = *E*_VBM_ − *E*_CBM_, the valence band maximum (VBM) edge potentials are calculated to be 1.25 V, 1.38 V and 1.37 V, respectively. Consequently, the band structure diagrams of the COFs were constructed, which reveal that all the COFs can thermodynamically drive photocatalytic H_2_O_2_ production.

**Fig. 2:**
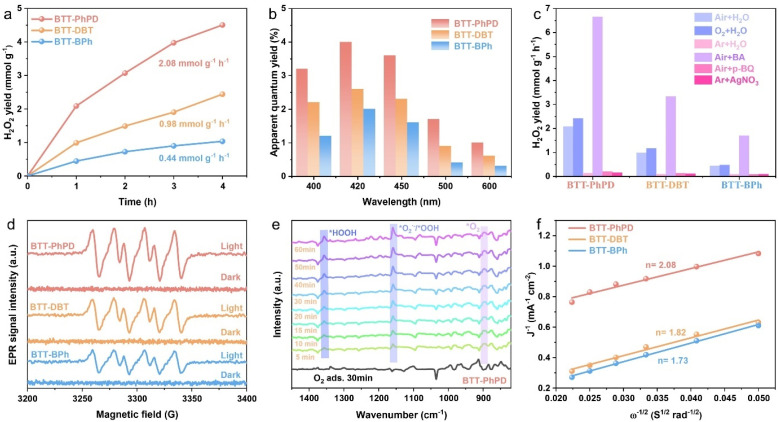
(a) Photocatalytic H_2_O_2_ production yields in pure water and air (*λ* > 420 nm); (b) wavelength-dependent AQY values; (c) capture experiment results; (d) EPR spectra tested with DMPO as the spin-trap agent; (e) typical *in situ* DRIFT spectra of BTT-PhPD during photocatalytic H_2_O_2_ production; (f) Koutecky–Levich plots recorded in the O_2_-saturated phosphate buffer solution.

The above structural characterization studies of the COFs demonstrate their highly ordered structures, large surface areas, abundant mesopores, and proper band structures, which are all suitable for photocatalytic H_2_O_2_ synthesis. Subsequently, their photocatalytic performances were systematically evaluated under simulated sunlight irradiation (*λ* > 420 nm) in pure water (without sacrificial agents) and air. As shown in Fig. 2a, compared with BTT-BPh (0.44 mmol g^−1^ h^−1^), the heteroatom-locked COFs, BTT-DBT (0.98 mmol g^−1^ h^−1^) and BTT-PhPD (2.08 mmol g^−1^ h^−1^), show much higher H_2_O_2_ production rates (2.1 and 4.7 times, respectively), which is comparable to that of most reported COF systems (Table S4). The corresponding apparent quantum efficiency (AQY) was also evaluated at various wavelengths from 400 to 600 nm (Fig. 2b),^63^ which shows that the COFs all possess the highest AQY at 420 nm, and BTT-PhPD has the highest AQY of 4.0% compared with BTT-DBT (2.6%) and BTT-BPh (1.9%). The solar-to-chemical conversion (SCC) efficiencies of the COFs are 0.12% (BTT-BPh), 0.16% (BTT-DBT) and 0.32% (BTT-PhPD), respectively. Besides, the photocatalytic performance of the COFs was also tested under an O_2_ atmosphere instead of air, which only displayed a little improvement in the H_2_O_2_ yield (typically from 2.08 to 2.42 mmol g^−1^ h^−1^ for BTT-PhPD, Fig. S9, summarized in Fig. 2c). When benzyl alcohol was used as the hole scavenger,^64,65^ the H_2_O_2_ yield of all the COFs increased nearly threefold (typically 2.08 to 6.65 mmol g^−1^ h^−1^ for BTT-PhPD, Fig. S10 and 2c). It can be seen that the H_2_O_2_ production rate consistently followed the order BTT-PhPD > BTT-DBT > BTT-BPh, which demonstrates the advantages of COFs with a heteroatom-locked acceptor in photocatalytic H_2_O_2_ production, especially when using N as the locking atom. Additionally, after the photocatalytic test, all the COFs still retain a certain degree of crystallinity (Fig. S11), and similar FT-IR spectra (Fig. S12), confirming their great structural stability. The H_2_O_2_ degradation experiments also indicated that the three COFs exhibited almost no degradation of H_2_O_2_ (Fig. S13). These all ensure a sustained COF-based photocatalytic H_2_O_2_ production.

Normally, photocatalytic H_2_O_2_ production may involve the oxygen reduction reaction (ORR) and water oxidation reaction (WOR), each of which also has several pathways.^66–68^ In order to investigate the specific reaction processes of these COF-based photocatalysts H_2_O_2_ production, a series of capture experiments, *in situ* characterization experiments, and electrochemical experiments were conducted. Under an argon (Ar) atmosphere, all the COFs produced negligible amounts of H_2_O_2_ (Fig. 2c), indicating that O_2_ is the key factor controlling H_2_O_2_ generation. When *p*-benzoquinone (*p*-BQ, 10 mM) was added as the superoxide radical (˙O_2_^−^) scavenger in pure water and air, a sharp decrease in H_2_O_2_ production rates was observed for all three COFs (typically from 2.08 to 0.05 mmol g^−1^ h^−1^ for BTT-PhPD, Fig. 2c). Additionally, in the nitroblue tetrazolium (NBT) experiment, the NBT content in the solution decreased to varying extents with prolonged illumination (Fig. S14), further confirming the generation of ˙O_2_^−^ during the reaction process.^69^ Furthermore, within the same time frame, the NBT content in the solution with BTT-PhPD decreased the most, demonstrating that BTT-PhPD generates more ˙O_2_^−^ than BTT-DBT and BTT-BPh, which could be the key to its high H_2_O_2_ yield. The production of ˙O_2_^−^ was also demonstrated by electron paramagnetic resonance (EPR) experiments, where COFs of the same mass exhibited clear DMPO–˙O_2_^−^ characteristic signals upon the addition of a spin trap (DMPO) under light irradiation, with BTT-PhPD showing the highest intensity (Fig. 2d). Diffuse reflectance infrared Fourier transform spectroscopy (DRIFTS) of BTT-PhPD in H_2_O vapor and O_2_ atmosphere further revealed the ORR reaction intermediates: the intensities of the O–O characteristic peak at 895 cm^−1^, the ˙O_2_^−^/˙OOH characteristic peak at 1155 cm^−1^, and the *HOOH characteristic peak at 1356 cm^−1^ all increase over time under illumination (Fig. 2e), further confirming the indirect ORR pathway for H_2_O_2_ production.^70,71^ Silver nitrate (AgNO_3_) was also used as the electron sacrificial agent to investigate the WOR half-reaction, but almost no H_2_O_2_ was detected in the AgNO_3_ solution (10 mM) under Ar atmosphere (Fig. 2c), suggesting that the WOR process contributes minimally to H_2_O_2_ production, and indicating that only the oxidation of H_2_O to O_2_ occurs in the WOR process. Furthermore, the reaction pathway for the 2e^−^ ORR can also be determined through electrochemical experiments on a rotating disk electrode (RDE). The linear sweep voltammetry (LSV) curves were obtained at various disk rotation speeds ranging from 400 to 2000 rpm (Fig. S15). Using the Koutecky–Levich method, the electron transfer numbers for the COFs were calculated to be 1.73 (BTT-BPh), 1.82 (BTT-DBT), and 2.08 (BTT-PhPD) (Fig. 2f).^72,73^ Based on the above results, it is inferred that the generation of H_2_O_2_ in the three COFs primarily involves indirect 2 e^−^ ORR (O_2_ + e^−^ → ˙O_2_^−^; ˙O_2_^−^ + e^−^ + 2H^+^ → H_2_O_2_) and 4 e^−^ WOR (2H_2_O − 4 e^−^ → O_2_ + 4H^+^) processes (typical schematic in Fig. S16).^74,75^

**Fig. 3:**
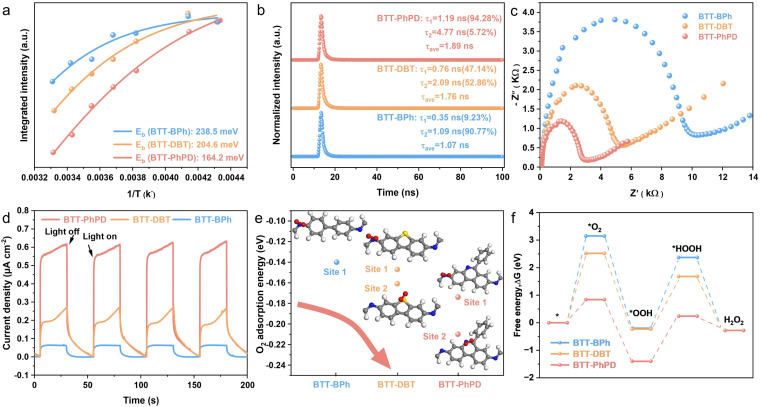
(a) Temperature-dependent integrated PL intensity and the extracted *E*_b_; (b) time-resolved PL spectra; (c) EIS spectra; (d) photocurrent response measurement under light irradiation (*λ* > 420 nm); (e) O_2_-adsorption energies at different sites (atom colour: N, blue; S, yellow; O, red; C, grey; H, white); and (f) Gibbs free energy diagrams of the ORR of the COFs.

To further explore the reasons for the heteroatom-lock effect on the promotion of photocatalytic H_2_O_2_ production, the differences of the fundamental photocatalytic processes between the three COFs were characterized systematically. First, temperature-dependent photoluminescence (PL) experiments were performed to investigate the charge carrier dynamics of the COFs. The emission intensity of all COFs decreases with increasing temperature, due to the progression of the thermally activated nonradiative recombination process under resonant excitation (Fig. S17).^76^ The calculated exciton binding energy (*E*_b_) results indicate that BTT-PhPD (164.2 meV) has a much lower *E*_b_ compared to BTT-DBT (204.6 meV) and BTT-BPh (235.8 meV) (Fig. 3a), suggesting that excitons in BTT-PhPD are more readily dissociated, facilitating their participation in photocatalytic reactions.^77^ Furthermore, time-resolved PL analysis in the nanosecond to microsecond time domain reveals that the fluorescence lifetimes of BTT-PhPD (1.89 ns) and BTT-DBT (1.76 ns) are nearly double that of BTT-BPh (1.07 ns) (Fig. 3b), suggesting a greater suppression of photogenerated carrier recombination in BTT-PhPD.^78^ Additionally, the semicircle radius in the electrical impedance spectra (EIS, Fig. 3c) gradually becomes smaller from BTT-BPh to BTT-DBT to BTT-PhPD, indicating the gradually lower charge transfer resistance. Besides, the photocurrent response test shows the gradually higher photocurrent density from BTT-BPh to BTT-DBT to BTT-PhPD (Fig. 3d), which also demonstrates more effective charge separation and migration. These data provide a physical explanation for why the COFs with heteroatom-locked acceptors exhibit higher photocatalytic H_2_O_2_ production efficiency: the heteroatom-locked acceptors extend the π-electron delocalization in COFs, which promotes the exciton separation, enhances the transport efficiency of photogenerated charge carriers, and consequently utilizes the absorbed solar energy better.

The above H_2_O_2_ reaction intermediate analyses prove the indirect 2 e^−^ ORR process of the three COF-based photocatalytic H_2_O_2_ production reactions (Fig. 2c–f), which means the interactions between the COFs and O_2_-related intermediate play an important role. DFT calculations were conducted to evaluate the oxygen adsorption energies at various sites of the three COFs (Fig. 3e and S18). It can be seen that the oxygen adsorption site of BTT-BPh is situated on the N atom of the imine bond; as for BTT-DBT and BTT-PhPD, the oxygen adsorption sites are located on the S atom of dibenzothiophene and the N atom of phenylpyridine, respectively, suggesting that the incorporation of heteroatoms has altered the O_2_ adsorption sites. Besides, the oxygen adsorption energy gradually enhances from BTT-BPh (−0.13 eV) to BTT-DBT (−0.16 eV) to BTT-PhPD (−0.21 eV), which confirms that the incorporation of the S/N heteroatom can effectively improve the oxygen adsorption capacity on the COF surface. Additionally, the changes in Gibbs free energies for the reaction intermediates on the COF surfaces were also simulated. As shown in Fig. 3f, the processes O_2_ → *O_2_ and *OOH → *HOOH exhibited positive free energy changes (the rate-determining steps), in which the Gibbs free energy gradually become lower from BTT-BPh (3.12 eV and 2.54 eV) to BTT-DBT (2.52 eV and 1.91 eV) to BTT-PhPD (0.84 eV and 1.54 eV); moreover, the energy barriers in the generation of other intermediates (*e.g.* *OOH and H_2_O_2_) also decreased from BTT-BPh to BTT-DBT to BTT-PhPD. These all indicate that the introduction of the N/S-heteroatom has enhanced the surface reaction kinetics of the ORR process toward H_2_O_2_ production.

**Fig. 4:**
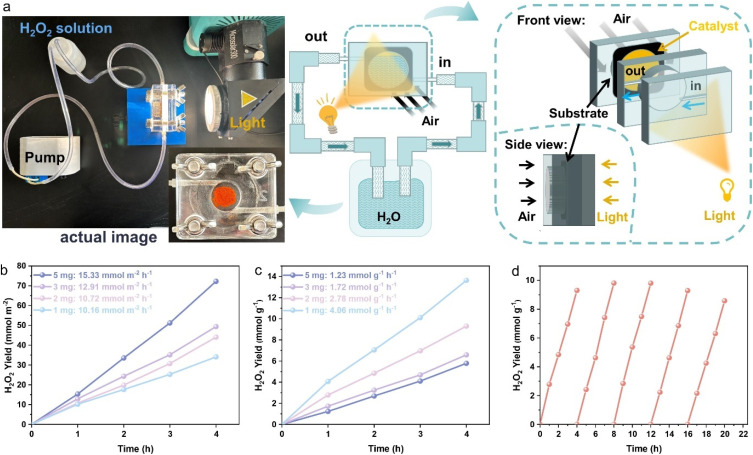
(a) Schematic diagrams and actual operating images of the gas diffusion system based on BTT-PhPD. (b and c) H_2_O_2_ yield relative to the reaction area and the mass of the supported catalyst, respectively, at different loading contents. (d) Cycling stability tests with a loading content of 2 mg.

As mentioned above, O_2_ mass transport is the first and a key step for the surface ORR, due to its low solubility in water (8 mg L^−1^ at 25 °C and 1 atm) and low diffusion coefficient (2.1 × 10^−5^ cm^−2^ s^−1^). Actually, O_2_ mass transport not only can be regulated through the structural modulation of the catalyst, but also can be controlled by the reaction system.^79^ Consequently, to further enhance the O_2_ diffusion efficiency, a gas diffusion system was introduced into the flow reaction system (Fig. 4a) instead of the above classic testing system (catalyst dispersed in water with stirring in an air atmosphere, Fig. S19) with BTT-PhPD (COF with the best photocatalytic H_2_O_2_ production performance) as the catalyst. Different amounts of the catalyst mixed with a binder were attached onto porous carbon paper (details in the SI), which was then placed between transparent quartz plates. Water was continuously flowing over one side of the catalyst-loaded carbon paper, driven by a peristaltic pump, while atmospheric oxygen entered through the other side and diffused into the COF surface to participate in the reaction. Upon illumination, atmospheric O_2_ and flowing H_2_O were catalysed by the COF to produce H_2_O_2_ at the three-phase interface, and the H_2_O_2_ solution was recycled and collected in a liquid storage bottle (Fig. 4a). The H_2_O_2_ production rates per reaction area and per unit mass were measured over 4 hours for catalyst loadings of 1 mg, 2 mg, 3 mg, and 5 mg. It was observed that as the loading amount decreased, the H_2_O_2_ production rate per unit reaction area slightly decreased (Fig. 4b), whereas the production rate per unit mass significantly increased (Fig. 4c). This may be due to the increased catalyst thickness at higher loadings, which impeded oxygen mass transport and reduced light utilization efficiency. The highest H_2_O_2_ production rate (4.06 mmol g^−1^ h^−1^) was achieved with 1 mg catalyst loading, showing a significant improvement *versus* the traditional system (Fig. S20). Furthermore, a decrease in the water flow rate (20 ml min^−1^ to 2 ml min^−1^) resulted in a slight enhancement in the photocatalytic H_2_O_2_ production efficiency (4.0 mmol g^−1^ h^−1^ to 4.7 mmol g^−1^ h^−1^) (Fig. S21). Additionally, the catalyst recycling process was very convenient, because the H_2_O_2_ solution in the storage bottle and the testing system could be easily replaced with fresh water for the next cycling experiment. As shown in Fig. 4d, the catalyst retained stable H_2_O_2_ production rate after five cycles, demonstrating the great stability of the reaction system. Consequently, compared to the traditional reaction system, the introduction of the gas diffusion system not only improves the O_2_ mass transport, but also realizes the immobilization and efficient recycling of the COF catalyst.

## Conclusions

To simultaneously improve the charge carrier separation and surface reaction efficiency in photocatalytic H_2_O_2_ production, a heteroatom-lock strategy is introduced into the acceptor structure of COFs, with the “lock” effect to enhance the coplanarity and conjugation, and the “heteroatom” effect to improve the O_2_ adsorption. It turns out that the photocatalytic H_2_O_2_ production yield (under pure water and air conditions) of the N-heteroatom locked BTT-PhPD (2.08 mmol g^−1^ h^−1^) is 2.1 times that of the S-heteroatom locked BTT-DBT (0.98 mmol g^−1^ h^−1^) and 4.7 times that of the original BTT-BPh (0.44 mmol g^−1^ h^−1^). Experimental results and theoretical calculations reveal that the higher H_2_O_2_ yield of the COFs with the heteroatom-locked acceptor is attributed to its lower exciton binding energy (*E*_b_) and smaller charge transfer resistance, together with the bigger O_2_ adsorption energy and lower transition state energy of the ORR intermediates. Additionally, a novel gas diffusion system is introduced to the reaction system to further improve the O_2_ mass transport, which not only enhances the photocatalytic H_2_O_2_ yield to 4.06 mmol g^−1^ h^−1^, but also realizes the immobilization and efficient recycling of the catalyst. This work elucidates the synergistic effect of specific COF units, and the reaction system toward improvement of the fundamental photocatalytic processes, providing valuable insights for the design of metal-free photocatalysts and reaction systems toward photocatalytic H_2_O_2_ production.

## Author contributions

Qianshuo Nan: methodology, data curation, formal analysis, writing – original draft. Jing Ning: software, data curation, formal analysis. Bing Han: data curation, investigation. Xuefeng Wang: methodology, investigation, funding acquisition. Ying-Ying Gu: investigation, formal analysis, funding acquisition. Shengxiang Zhou: investigation. Hongtao Wei: investigation. Guangqiang Cao: investigation. Xuehui Li: investigation. Guangze Zhang: investigation. Yonggang Jia: software, investigation, funding acquisition. Long Hao: supervision, project administration, conceptualization, resources, writing – review & editing, funding acquisition.

## Conflicts of interest

The authors declare no competing financial interest.

## Supplementary Material

SC-OLF-D5SC05346C-s001

## Data Availability

All the data supporting this article have been included in the main text and the supplementary information (SI). Supplementary information: the reagents required, the operation procedures and the figures of the relevant experiments. See DOI: https://doi.org/10.1039/d5sc05346c.
