# Supplementary material for: Boosting the photocatalytic H2O2 production of covalent organic frameworks with a heteroatom-locked acceptor and gas diffusion system
Source: Chem Sci. 2025 Nov 8;17(1):466–74. doi: 10.1039/d5sc05346c (PMC12621264; doi:10.1039/d5sc05346c)
Supplement: SC-017-D5SC05346C-s001 [file SC-017-D5SC05346C-s001.pdf]

Supplementary Information (SI) for Chemical Science.
This journal is © The Royal Society of Chemistry 2025

Supporting Information

Boosting Photocatalytic H_2O_2 Production of Covalent Organic Framework with Heteroatom-Locked Acceptor and Gas Diffusion System

Qianshuo Nan, † Jing Ning, † Bing Han, Hongtao Wei, Xuefeng Wang, Ying-Ying Gu,* Shengxiang Zhou, Guangqiang Cao, Guangze Zhang, Xuehui Li, Yonggang Jia,* and Long Hao**

Q. Nan, Y. Y. Gu
College of Chemistry and Chemical Engineering, China University of Petroleum (East China), Qingdao 266580, P. R. China.
E-mail: yingyinggu@upc.edu.cn

Q. Nan, J. Ning, H. Wei, X. Wang, G. Cao, G. Zhang, X. Li, L. Hao
College of Chemistry and Pharmaceutical Sciences, Qingdao Agricultural University, No.700 Changcheng Road, Qingdao 266109, P. R. China
E-mail: wxf@qau.edu.cn; haol@qau.edu.cn
† Q. Nan and J. Ning contributed equally to this work

B. Han
MOE Key Laboratory of Resources and Environmental Systems Optimization, College of Environmental Science and Engineering, North China Electric Power University, Beijing, 102206, P.R. China

S. Zhou
Marine Science Research Institute of Shandong Province, 7 Youyun Road, Qingdao, 266104, China

Y. Jia
Shandong Provincial Key Laboratory of Marine Environment and Geological Engineering, Key Laboratory of Marine Environment and Ecology, Ocean University of China, Qingdao 266100, China
Email: yonggang@ouc.edu.cn

Section 1. Materials and methods

Materials: 4,4'-diaminobiphenyl (BPh), 3,7-Diaminodibenzothiophene (DBT), 6-Phenylphenanthridine-3,8-diamine (PhPD), 5,5-Dimethyl-1-pyrroline N-oxide (DMPO) and p-benzoquinone (p-BQ) were purchased from Shanghai bidepharm Co., Ltd. p-Dithiane-2,5-diol, 1,2-dichlorobenzene (o-DCB), 1-butanol (n-BuOH), tetrahydrofuran (THF), N, N-dimethylformamide (DMF), nitro-blue tetrazolium (NBT), Pyridinium Chlorochromate (PCC) and triethylamine (TEA) were purchased from Energy Chemical. Iron powder (Fe), bromine (Br_2), 1,3,5-trimethylbenzene, Chloroform ($CHCl_3$), Potassium Acetate (KOAc), Potassium Hydroxide (KOH), Acetic acid (AcOH), Silver nitrate ($AgNO_3$), Potassium iodide (KI), Barium Sulfate ($BaSO_4$), Cerium(IV) Sulfate ($Ce(SO_4)_2$), Hydrogen Peroxide (H_2O_2, ~30%), Potassium Dihydrogen Phosphate (KH_2PO_4) and Dipotassium Hydrogen Phosphate (K_2HPO_4), Potassium Chloride (KCl) and Sodium Sulfate (Na_2SO_4) were purchased from Sinopharm Co.. Nafion® D-521 dispersion (5% w/w in water and 1-propanol) was purchased from Alab (Shanghai) Chemical Technology Co., Ltd. The other common reagents and solvents were commercially available and used without further purification.

Characterizations: Power X-ray diffraction (PXRD) patterns were collected on a D8 Advance diffractometer in reflection geometry operating with a Cu Kα anode (λ = 1.54178 Å) at 40 kV and 40 mA and with a slit width of 0.1 mm. The attenuated total reflection Flourier transformed infrared (FT-IR) spectra of the samples were collected on a Nicolet iS10

spectrometer. Field emission transmission electron microscope (FE-TEM) images were got from a Tecnai G2 F20 U-TWIN microscope. Nitrogen adsorption/desorption isotherm measurements at 77 K were carried out with an ASAP 2020 plus HD88 analyzer, and the Brunauer-Emmett-Teller (BET) method and density functional theory (DFT) pore model were utilized to calculate the specific surface areas and pore size distributions, respectively. Electron paramagnetic resonance (EPR) measurements were recorded on the Bruker A-300 instrument with DMPO as spin-trapping agent for the detection of superoxide radical ($\cdot O_2^-$). The photocatalysts (2 mg) were well dispersed in a O_2-saturated water/methanol (1:1, 500 μL) containing DMPO (0.1 mmol) and a 300 W Xenon lamp was applied as the light source. UV-vis diffuse reflectance absorption spectra (DRS) were recorded on a Hitachi-UV-3900 spectrophotometer with $BaSO_4$ as reference. Steady-state photoluminescence (PL) spectra were recorded on a Hitachi F-7000. Temperature-dependent PL spectra and time-resolved PL spectra were obtained on Edinburgh Instruments FLS-1000 spectrophophotometer. Thermogravimetry (TG) curves were recorded in nitrogen on a TGA-1000B analyzer. For in-situ diffuse reflectance infrared Fourier transfer (DRIFT) spectra, the photocatalyst was put inside the reaction cell consisting of a sample cup and a cover dome. During the measurement, the photocatalyst powder was pretreated with Ar flow for 10 min. Water vapor was introduced to the reaction cell by oxygen flow for 10 min. After reaching vapor adsorption equilibrium, a Xe lamp was used to irradiate the photocatalyst, and the IR spectra were collected at intervals.

Photocatalytic H_2O_2 Production with Classic Testing System: typically, 5 mg of the COF was dispersed in 50 mL of distilled water (When benzyl alcohol was used as sacrificial agent, 5 mL of benzyl alcohol and 45 mL of water mixture were used instead) via ultrasonication for 20 min. The suspension was irradiated by a 400 W xenon lamp (Beijing Perfect light, PLS-SME400E H1) equipped with a 420 nm long-pass filter (When creating an O_2-enriched environment, O_2 was pumped into the suspension in dark environment for 30 minutes before starting the illumination). The reaction was kept at 25 °C by a flow of cooling water (Fig. S17). 1 mL of the suspension was collected every 1 h and filtered through a 0.22 μm PTFE membrane filter to remove the photocatalyst. The concentration of H_2O_2 was determined by the iodometry method. Specifically, 1 mL of solution was added to the mixture of 2 mL of 0.4 M potassium iodide (KI) and 1 mL of 0.1 M potassium hydrogen phthalate ($C_8H_5KO_4$), which was kept at 35 °C for 1 h before testing the ultraviolet absorption. Under acidic conditions, H_2O_2 can react with I^- to generate I_3^- ($H_2O_2 + 3\ I^- + 2\ H^+ \rightarrow I_3^- + 2\ H_2O$), which exhibits strong ultraviolet absorption at ~352 nm. The linear relationship between H_2O_2 concentration and the absorption intensity is established as below.

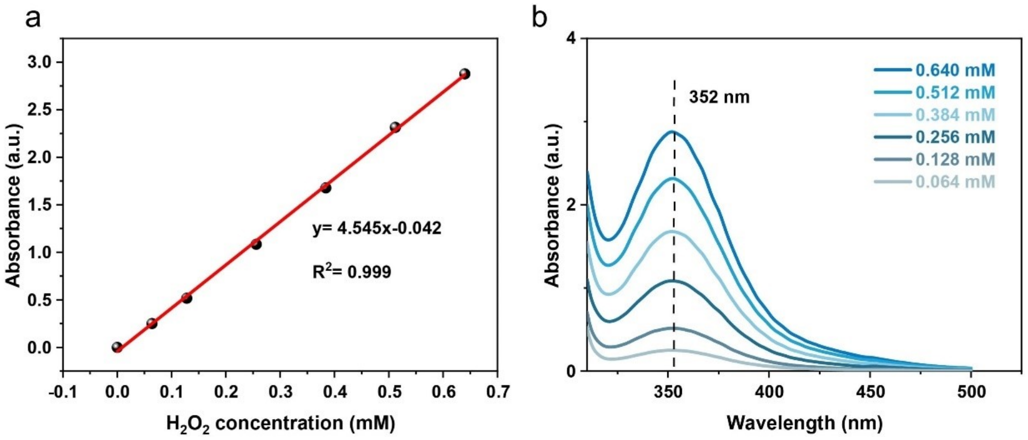

Apparent quantum yield (AQY) measurements: The AQY for H_2O_2 was measured under the irradiation of a 400 W Xenon lamp equipped with different bandpass filters (400, 420, 450, 500 and 600 nm). After ultrasonication under Air atmosphere, the photocatalytic reaction was conducted in pure water (50 mL) with photocatalyst (30 mg) at 25 °C. The AQY is calculated by the following equation:[1]

$$\mathrm{AQY}\ (\%) = \frac{2n_{(H_2O_2)}}{n_{\mathrm{photon}}} \times 100\% \quad (1)$$

$$n_{\mathrm{photon}} = \frac{E_{\mathrm{total}}}{E_{\mathrm{photon}}} = \frac{\mathrm{ISt}}{N_A hc/\lambda} \quad (2)$$

Where $n_{(H_2O_2)}$ is the amount of H_2O_2 generated (mol), n_{photon} is the photon number entered into the photoreactor (mol), N_A is Avogadro constant (6.022 × 1023 mol^{-1}), h is the Planck constant (6.626 × 10^{-34} J s), c is the speed of light (3 × 108 m s^{-1}), S is the irradiation area (1.0 cm^2), I is the intensity of irradiation light, t is the photoreaction time (3600 s), λ is the wavelength of the monochromatic light (nm).

Solar-to-chemical energy conversion (SCC) efficiency measurements: For the evaluation of SCC efficiency, photocatalytic H_2O_2 production experiments were conducted at 25 °C in pure water (50 mL) with photocatalysts (30 mg), using an AM 1.5 G solar simulator as the light source. The SCC efficiency is calculated by the following equation:

$$\mathrm{SCC}\ (\%) = \frac{\Delta G_{H_2O_2} \times n_{H_2O_2}}{E_{\mathrm{tatol}} \times t} \times 100 = \frac{\Delta G_{H_2O_2} \times n_{H_2O_2}}{\mathrm{IST}} \times 100 \quad (3)$$

where $\Delta G_{H_2O_2}$ is the free energy for H_2O_2 generation (117 kJ mol^{-1}), I is the light power

intensity, S is the irradiation area (1.0 cm^2), t is the photoreaction time (3600 s).

Control experiments: To investigate the reaction mechanism and intermediates, photocatalytic H_2O_2 reactions were performed under different conditions and with different sacrificial reagents. 5 mg catalyst was added to p-benzoquinone (p-BQ, superoxide radical scavenger, 10 mM) under Air atmosphere, $AgNO_3$ (e^- scavenger, 10 mM) under Ar atmosphere respectively, to conduction control experiments. When using $AgNO_3$ solution, to avoid the interference of AgI formed by the reaction between $AgNO_3$ and KI, the cerium (IV) sulfate titration method is used to detect the generation of H_2O_2 according to the previous reported method. $Ce(SO_4)_2$ solution (1 mM) was prepared as an oxidizing agent to reduce H_2O_2 ($2\ Ce^{4+} + H_2O_2 \rightarrow 2\ Ce^{3+} + 2\ H^+ + O_2$). The concentration of Ce^{4+} before and after the reaction was measured by UV-vis spectroscopy to calculate the amount of H_2O_2 produced, followed the equation as below:

$$n_{(H_2O_2)} = \frac{1}{2} \times n_{(Ce^{4+})} \quad (4)$$

The linear relationship between Ce^{4+} concentration and the absorption intensity was established.

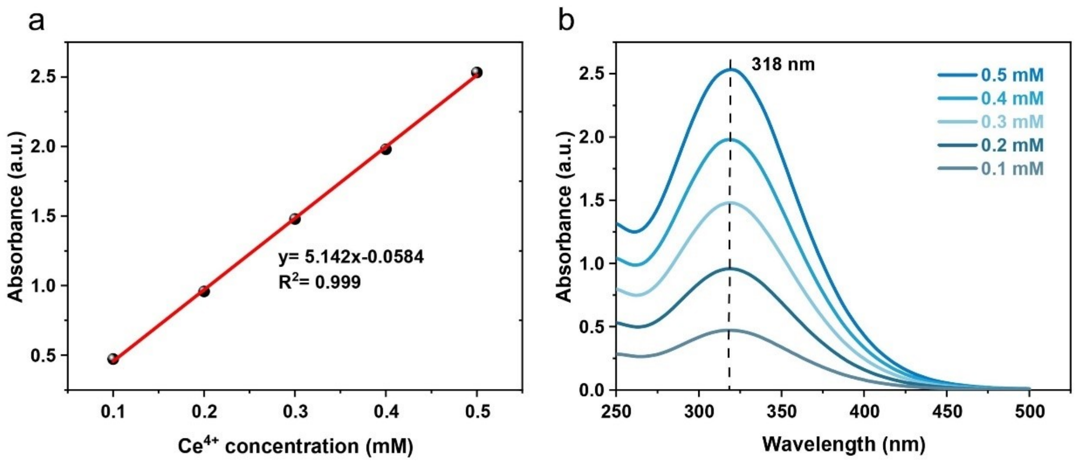

a
Absorbance (a.u.)
y= 5.142x-0.0584
R2= 0.999
Ce4+ concentration (mM)
b
318 nm
0.5 mM
0.4 mM
0.3 mM
0.2 mM
0.1 mM
Absorbance (a.u.)
Wavelength (nm)

NBT experiments: Specifically, 20 mg sample powders were dispersed into 50 mL nitro-blue tetrazolium (NBT) aqueous solution. The so-formed suspensions were stored in dark for 30 min to reach desorption-adsorption equilibrium. Subsequently, the suspensions were illuminated by visible light ($\lambda > 420$ nm) for 1.5 h under Air atmosphere. An aliquot of 2.5 mL was collected at a 30 min interval which was then filtered using a 0.22 μm filter membrane. The production of superoxide radicals is detected by observing the intensity change at 259 nm in the ultraviolet-visible spectrum of the NBT solution.

Photocatalytic H_2O_2 Production with Gas Diffusion System: Before the reaction, BTT-PhPD with different masses (1 mg, 2 mg, 3 mg, 5 mg) were dissolved in 200 μL of isopropanol, mixed with 5 wt.% Nafion solution (20 μL), and sonicated for 30 minutes. The mixture was then drop-coated onto hydrophobic carbon paper (Model: GP02P), which was dried at 60 °C in an oven overnight. During the following photocatalytic testing, the catalyst-coated carbon paper was positioned between transparent quartz plates, and water was continuously flowing over one side of the catalyst-loaded carbon paper, driven by a peristaltic pump, while atmospheric oxygen entered through the other side and diffused into the COF surface to participate the reaction (see schematic and digital photo in Figure 4a). The peristaltic pump circulated 50 mL of water from the storage bottle over the catalyst surface at a flow rate of 10 mL/min. At regular intervals, 1 mL of solution from the storage bottle were collected and the H_2O_2 concentration was measured via iodometric titration. For the cyclic experiment, after one cycle, the H_2O_2

solution in the storage bottle and the testing system was replaced with fresh water before the next cycling experiment.

Photocatalytic decomposition of H_2O_2 by the COFs： The decomposition of H_2O_2 was conducted by suspending catalysts (5 mg) in aqueous solution (50 mL) containing H_2O_2 (2 mM) under Ar atmosphere. A 400 W Xe lamp (Beijing Perfect light, PLS-SME400E H1) was used as the light source. A cutoff filter (CEL-UVIRCUT420) was used to achieve visible-light irradiation (420 nm < λ).

Rotating disk electrode (RDE) measurements: To prepare the working electrode for RDE measurements, 2 mg of the COF was dispersed in 400 μL of EtOH containing 4 μL of Nafion (5 wt. %) by 30 minutes ultrasonication. Then, 10 μL of the slurry was dripped onto a glassy carbon rotating disk electrode and dried at room temperature. In this measurement, the as-prepared RDE, a platinum plate, and an Ag/AgCl were used as the working electrode, counter electrode, and reference electrode, respectively. The pathway of O_2 reduction reaction (ORR) was investigated by RDE in an O_2 saturated 0.1 M phosphate buffer solution (pH = 7). The linear sweep voltammograms (LSV) were obtained under room temperature with a scan rate of 5 mV s^{-1} and different rotating speeds ranging from 400 rpm to 2000 rpm. The average number of electrons (n) involved in the ORR of is obtained by the linear regression of the plots using the following equation:[2]

$$j^{-1} = j_k^{-1} + B^{-1} \times \omega^{-1/2} \quad (5)$$

$$B = 0.2nF\nu^{-1/6}CD^{-2/3} \quad (6)$$

Where j is the measured current density, j_k is the kinetic current density, ω is the rotating speed (rpm), F is the Faraday constant (96485 C mol^{-1}), ν is the kinetic viscosity of water (0.01 cm^2 s^{-1}), C is the bulk concentration of O_2 in water (1.26×10^{-3} mol cm^{-3}), and D is the diffusion coefficient of (2.7×10^{-5} cm^2 s^{-1}), respectively.

Electrochemical measurement: Electrochemical measurements were conducted with the Chi760e electrochemical workstation from Chenhua Instrument. A traditional three-electrode system was used with COF-based working electrode, platinum wire as the counter electrode and Ag/AgCl (saturated KCl) as reference electrode. The electrolyte was 0.2 M Na_2SO_4 aqueous solution (pH 7). COFs powder (4 mg grinded in an agate mortar before use) was mixed with 40 μL of Nafion and 150 μL of ethanol, ultra-sonicated for 30 min to obtain a slurry. The suspension was spread onto the surface of indium-tin oxide (ITO) glass (1 cm × 1 cm), forming a film after air drying. Then the photocurrent measurements were performed with a 400 W Xe lamp (Beijing Perfect light, PLS-SME400E H1) with a 420 nm cut-off filter and the electrochemical impedance spectroscopy (EIS) curves were recorded at 10^{-1} to 10^5 Hz, the initial potential is 2 V. The MS plots were obtained at frequencies of 1.0, 1.5, and 2.0 kHz. For photocurrent response, the light on/off photocurrent response was recorded with time interval of 25 s.

Photoluminescence (PL) measurement: Temperature-dependent photoluminescence spectra were recorded by Edinburgh Instruments (FLS1000) to determine the exciton binding

energy (E_b) of COFs. The intensity of PL decreases when the temperature increases. The corresponding E_b is calculated through fitting the intensity data with Arrhenius equation:[3]

$$I(T)/I_0 = \frac{1}{1 + Ae^{-E_b/(k_B T)}} \quad (7)$$

where $I(T)$ represents the normalized integrated PL intensity and I_0 is the value at 0 K. E_b is the binding energy. A is a proportional constant and k_B is the Boltzmann constant.

Time-resolved PL decays were fitted by a biexponential model:

$$I_{(TRPL)}(t) = A_1 e^{-t/\tau 1} + A_2 e^{-t/\tau 2} \quad (8)$$

$$\tau_{ave} = \frac{\tau_1 Rel_1 + \tau_2 Rel_2}{100} \quad (9)$$

$$Rel_n = \frac{A_n \tau_n}{A_1 \tau_1 + A_2 \tau_2} \times 100, n = 1, 2 \quad (10)$$

where $I_{TRPL}(t)$ is the intensity of TRPL signal, A_1 and A_2 are the amplitudes, τ_1 and τ_2 are the lifetimes, τ_{ave} is the intensity average lifetime, $Rel_n$is the fractional intensity of that component.

Computational details: All density functional theory calculations based on the first-principles were performed with DMOL3 package of Materials Studio. The exchange-correlation potential was described via using the generalized gradient approximation of Perdew-Burke-Ernzerhof (GGA-PBE). Grimme method was used for DFT-D correction. Convergence was reached when the total energies converge within 10^{-5} Ha, and the convergence threshold of force within 0.002 Ha/Å. The total energy was calculated using the self-consistent field iteration (SCF) method, and 0.003 Ha smearing was used. The Monkhorst-Pack grid was used for Brillouin k-point sampling, with a grid size of 3×3×1. The adsorption energy (E_{ads}) was

calculated based on:

$$E_{ads} = E_{system} - E_{catalyst} - E_{O_2} \quad (11)$$

where E_{system}, $E_{catalyst}$, and E_{O_2} are the total energy of the optimized system with adsorbed O_2, the isolated catalyst, and O_2, respectively.

The Gibbs free energy was calculated by the following formula:

$$\Delta G=\Delta E+\Delta ZPE-T\Delta S \quad (12)$$

where ΔE is the electronic energy, ΔZPE and ΔS are the zero-point energy difference and entropy change between the products and reactants, respectively, and T is the Kelvin temperature (298.15 K). The photocatalytic process of ORR was simulated as following reactions:

$$*+O_2 \rightarrow *O_2 \quad (13)$$

$$*O_2+H^++e^- \rightarrow *OOH \quad (14)$$

$$*OOH+H^++e^- \rightarrow *HOOH \quad (15)$$

Section 2. Synthesis of the monomer BTT and the corresponding COFs

Synthesis of benzo[1,2-b:3,4-b':5,6-b'']trithiophene-2,5,8-tricarbaldehyde (BTT)

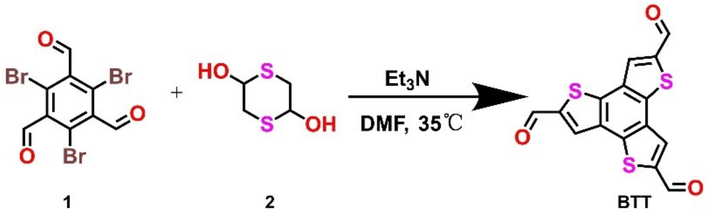

BTT was synthesized based on our previous work with some modifications.[4,5] In brief, 2,4,6-tribromobenzene-1,3,5-tricarbaldehyde (1, prepared according to the literature [6], 350 mg, 0.87 mmol) and p-dithiane-2,5-diol (2, 220 mg, 1.44 mmol) were dispersed into 10 mL DMF in a three-necked flask at 273 K, then triethylamine (0.73 mL, 5.24 mmol) was added, and the reaction system was gradually heated to 35 °C, and stirred for 12 h. Then, the reaction mixture was poured into water, and the precipitate was centrifuged and washed constantly with water and THF, respectively, yielding the dark yellow BTT (173 mg, 60%).

Synthesis of benzo[1,2-b:3,4-b':5,6-b'']trithiophene-2,5,8-triyltrimethanol. (2)

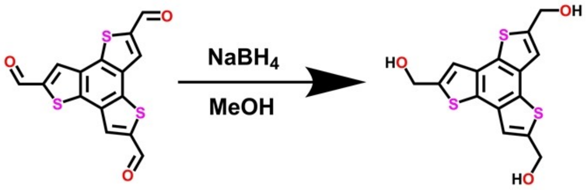

Owing to the exceedingly poor solubility of BTT, obtaining its ^{1}H NMR spectrum was impractical. We employed an indirect method to verify its structure[5]: Typically, to a suspension of BTT (0.165 g, 0.5 mmol) in methanol (30 mL), $NaBH_4$ (50 mg, 1.32 mmol) was added in small portions. The mixture was stirred for 24 h under reflux. After cooling down, the resulting mixture was poured into water and the precipitate was collected by filtration. After washed with water and cold methanol and dried at 60°C under reduced pressure, 0.161 g (96%) of compound 2 was obtained, as benzo[1,2-b:3,4-b':5,6-b'']trithiophene-2,5,8-triyltrimethanol. ^{1}H NMR (500 MHz, DMSO-d6) δ 7.53 (s, 3H), 5.75 (t, J = 5.9 Hz, 3H), 4.83 (d, J = 5.8 Hz, 6H). ^{13}C NMR (126 MHz, DMSO-d6) δ 147.61, 131.18, 130.09, 118.84, 59.28.)

Synthesis of BTT-BPh

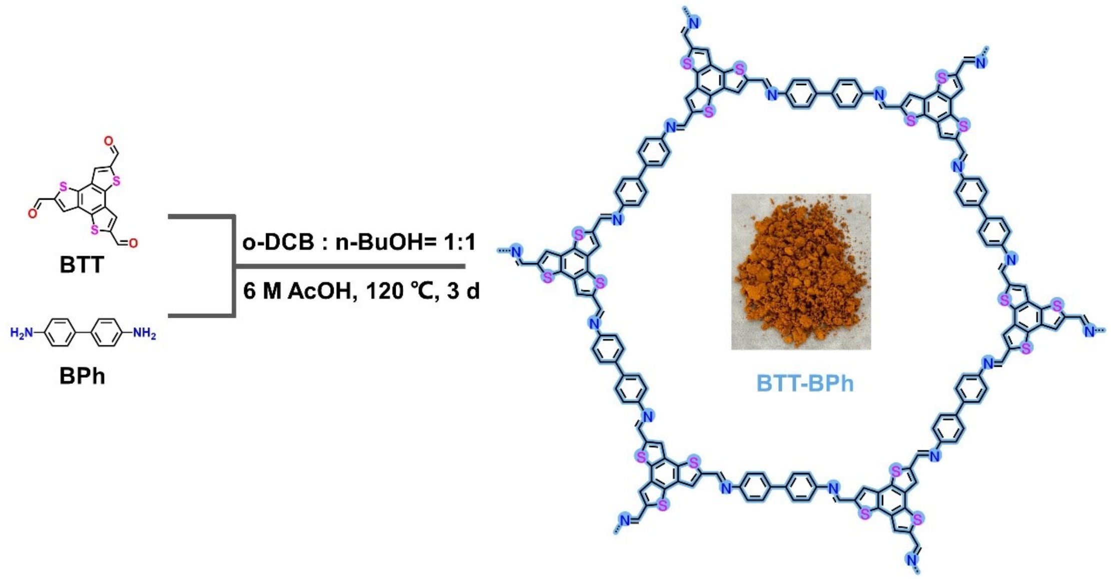

an 80 mL Pyrex tube was charged with BTT (99.1 mg, 0.3 mmol), 4,4'-diaminobiphenyl (BPh) (82.9 mg, 0.45 mmol), 1,2-dichlorobenzene (o-DCB, 2.25 mL), 1-butanol (n-BuOH, 2.25 mL), and 6 M acetic acid (AcOH, 450 μL). After sonicated for 10 minutes and degassed by freeze-pump-thaw cycles for three times, the tube was sealed under vacuum, and heated at 120 °C for 72 h without any disturbance. After cooled down to room temperature, the solid product was collected by centrifugation, washed constantly with tetrahydrofuran (THF) until the supernatant was clear, and dried in the vacuum oven at 60 °C overnight to get BTT-BPh (dark yellow color, 151 mg, 91%).

Synthesis of BTT-DBT

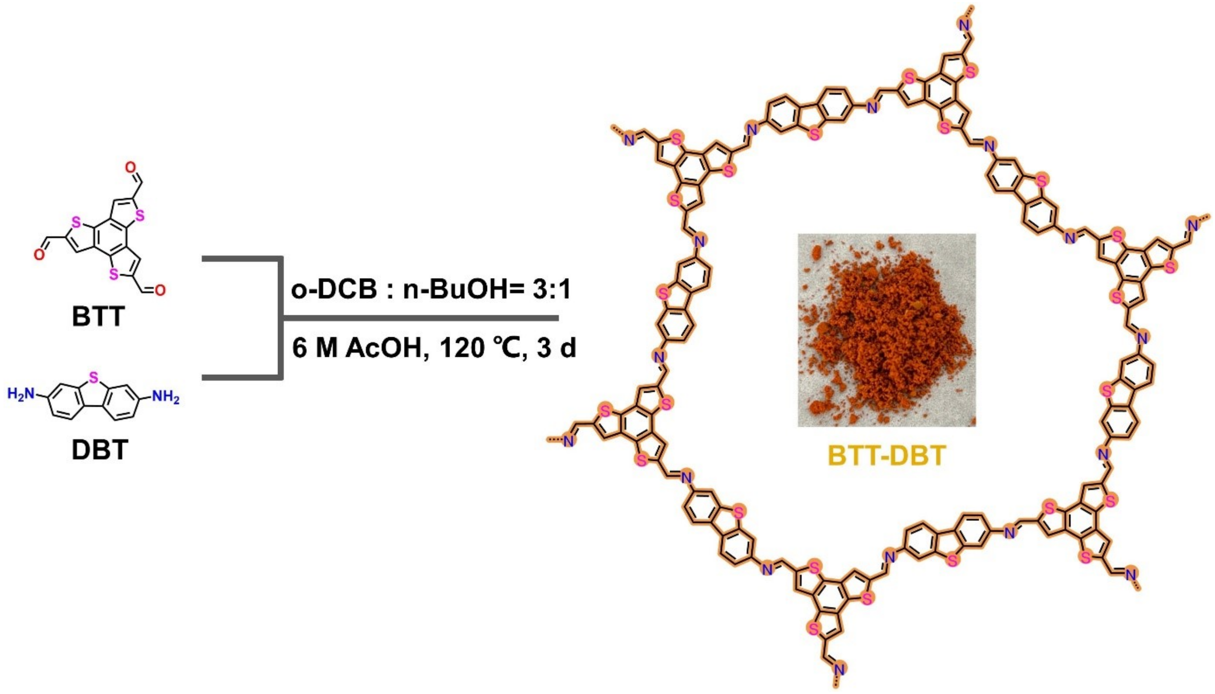

an 80 mL Pyrex tube was charged with BTT (99.1 mg, 0.3 mmol), 3,7-Diaminodibenzothiophene (DBT) (96.5 mg, 0.45 mmol), 1,2-dichlorobenzene (o-DCB, 3.375 mL), 1-butanol (n-BuOH, 1.125 mL), and 6 M acetic acid (AcOH, 450 μL). After sonicated for 10 minutes and degassed by freeze-pump-thaw cycles for three times, the tube was sealed under vacuum, and heated at 120 °C for 72 h without any disturbance. After cooled down to room temperature, the solid product was collected by centrifugation, washed constantly with tetrahydrofuran (THF) until the supernatant was clear, and dried in the vacuum oven at 60 °C overnight to get BTT-DBT (orange red color, 162 mg, 93%).

Synthesis of BTT-PhPD

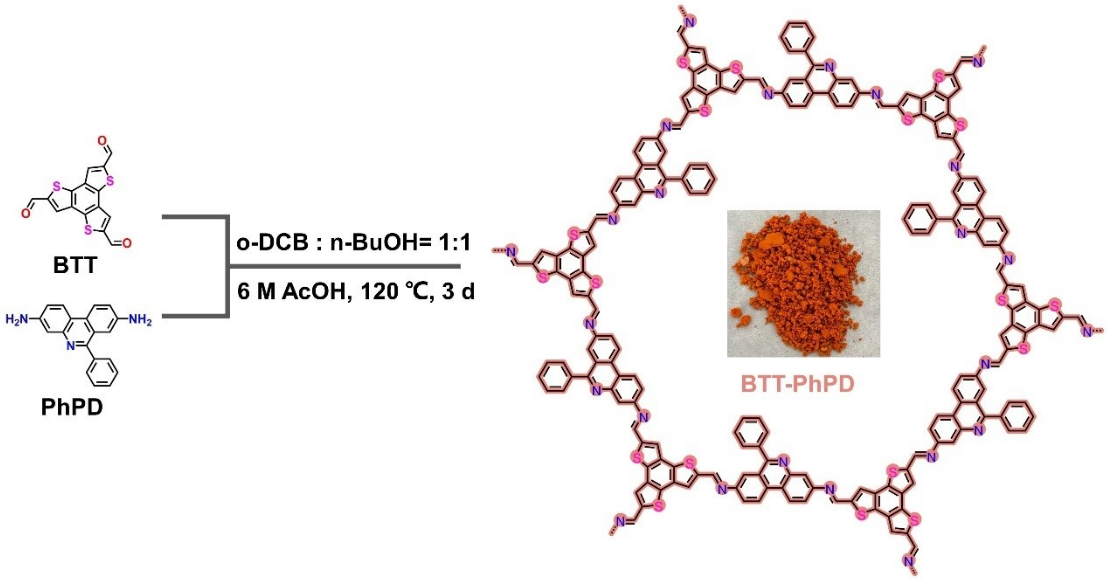

an 80 mL Pyrex tube was charged with BTT (99.1 mg, 0.3 mmol), 6-phenylphenanthridine-3,8-diamine (PhPD) (128.4 mg, 0.45 mmol), 1,2-dichlorobenzene (o-DCB, 2.25 mL), 1-butanol (n-BuOH, 2.25 mL), and 6 M acetic acid (AcOH, 450 μL). After sonicated for 10 minutes and degassed by freeze-pump-thaw cycles for three times, the tube was sealed under vacuum, and heated at 120 °C for 72 h without any disturbance. After cooled down to room temperature, the solid product was collected by centrifugation, washed constantly with tetrahydrofuran (THF) until the supernatant was clear, and dried in the vacuum oven at 60 °C overnight to get BTT-PhPD (orange color, 190 mg, 90%).

Section 3. Supplementary figures and discussion

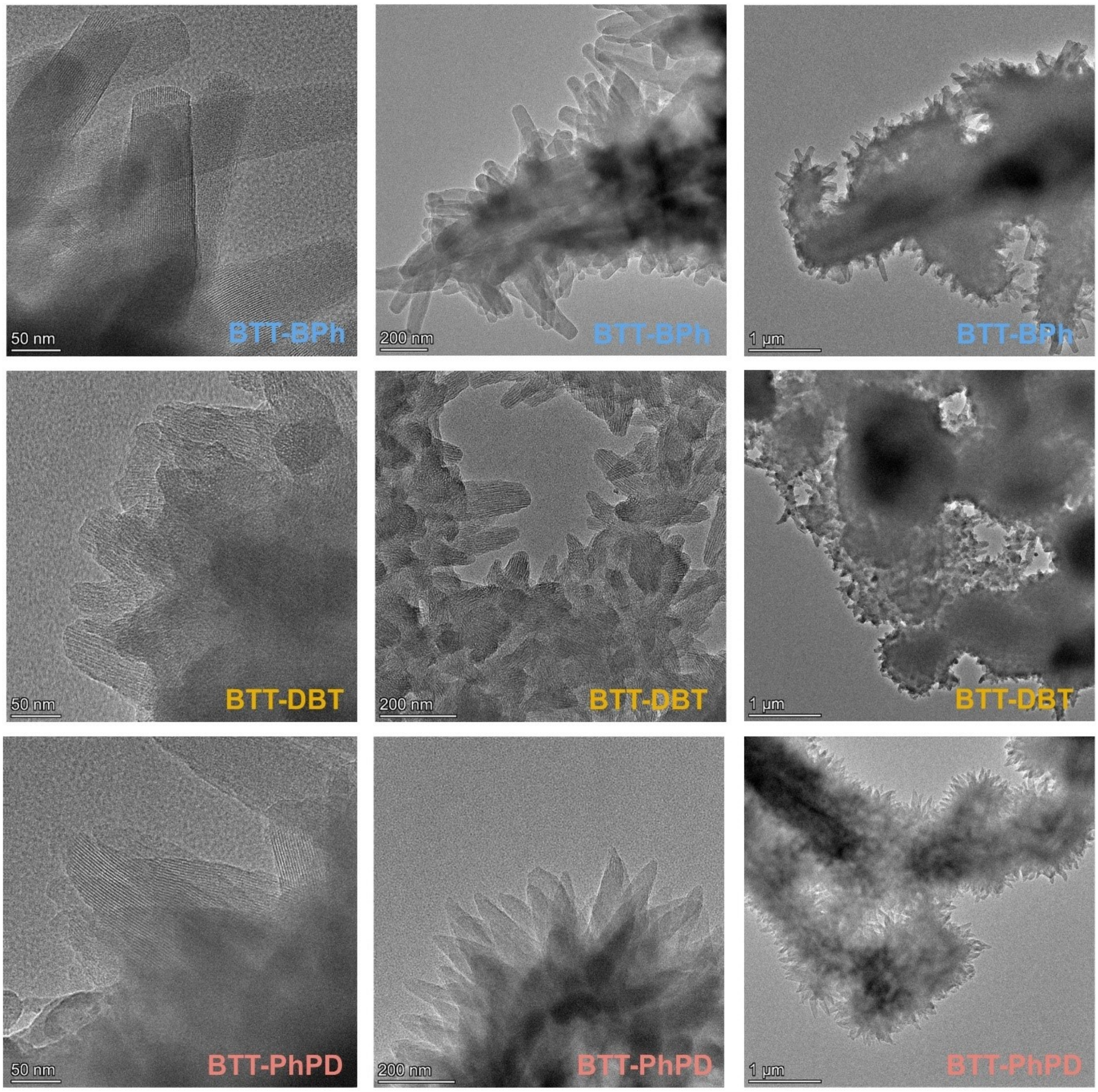

Fig. S1. Typical TEM images of the COFs.

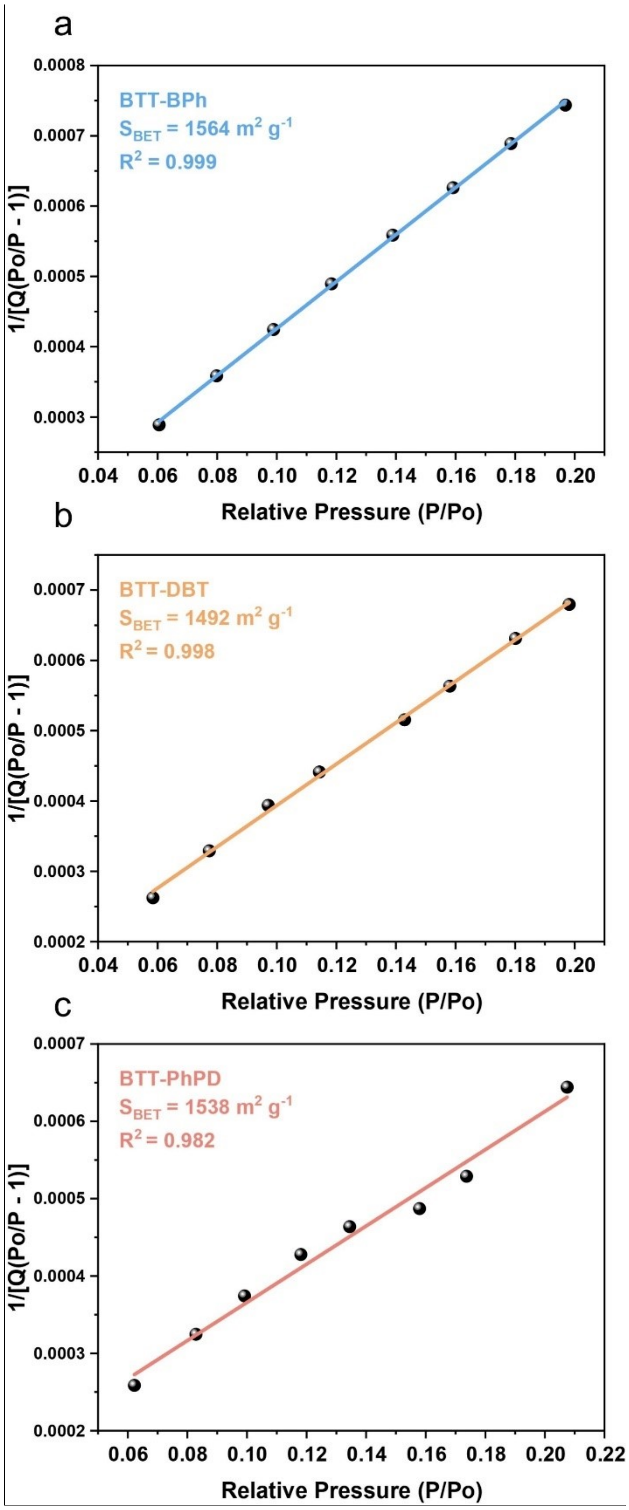

Fig. S2. BET Surface area plots of the COFs.

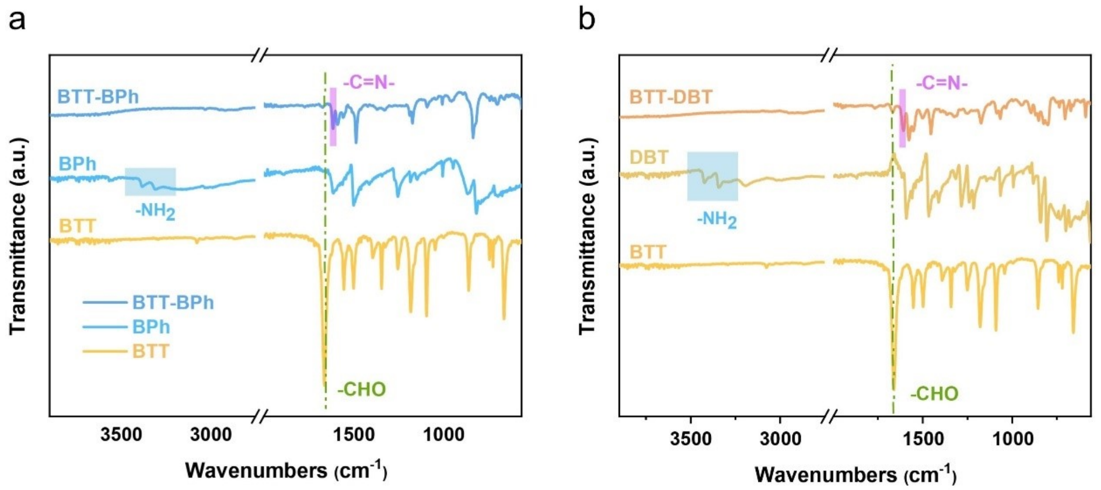

Fig. S3. FT-IR spectra of (a) BTT-BPh, BPh and BTT and (b) BTT-DBT, DBT and BTT.

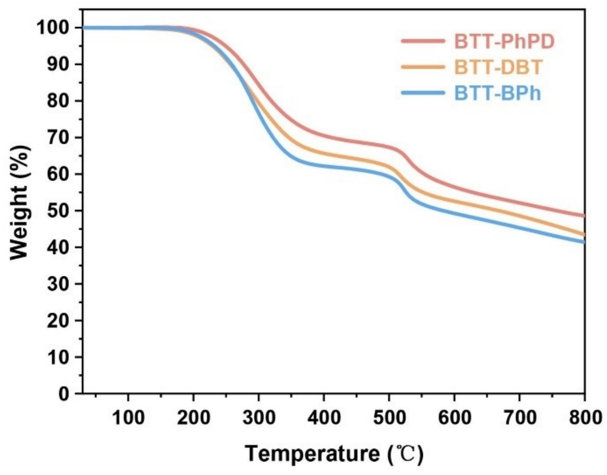

Fig. S4. TGA curves of the COFs.

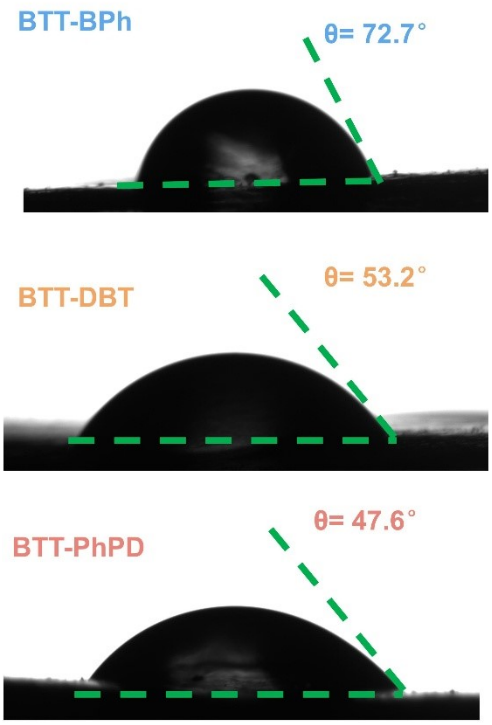

Fig. S5. Water contact angle of the COFs

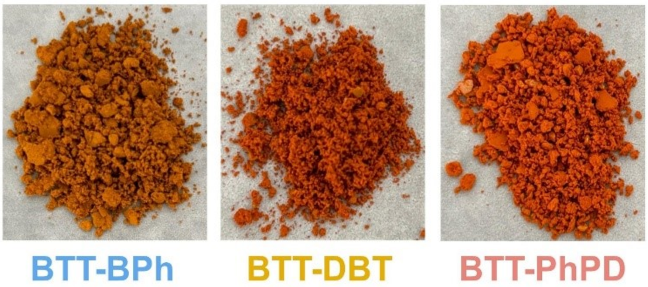

Fig. S6. digital photos of the COFs.

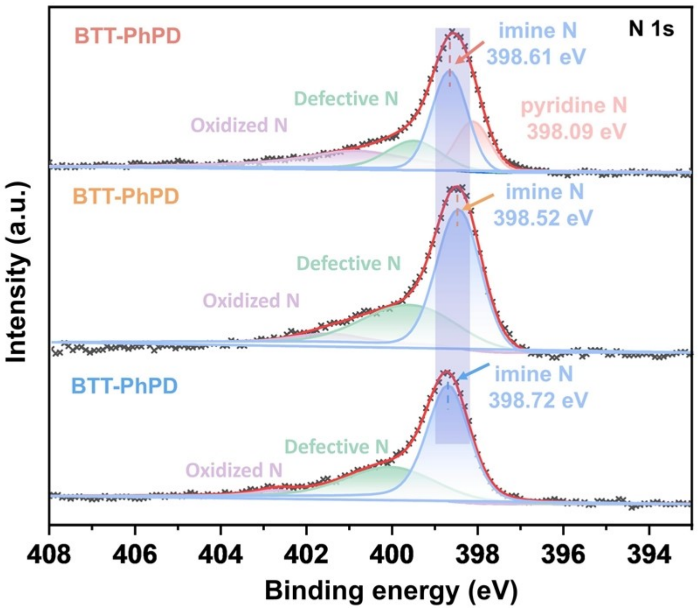

Figure S7. The N1s XPS spectra of the COFs

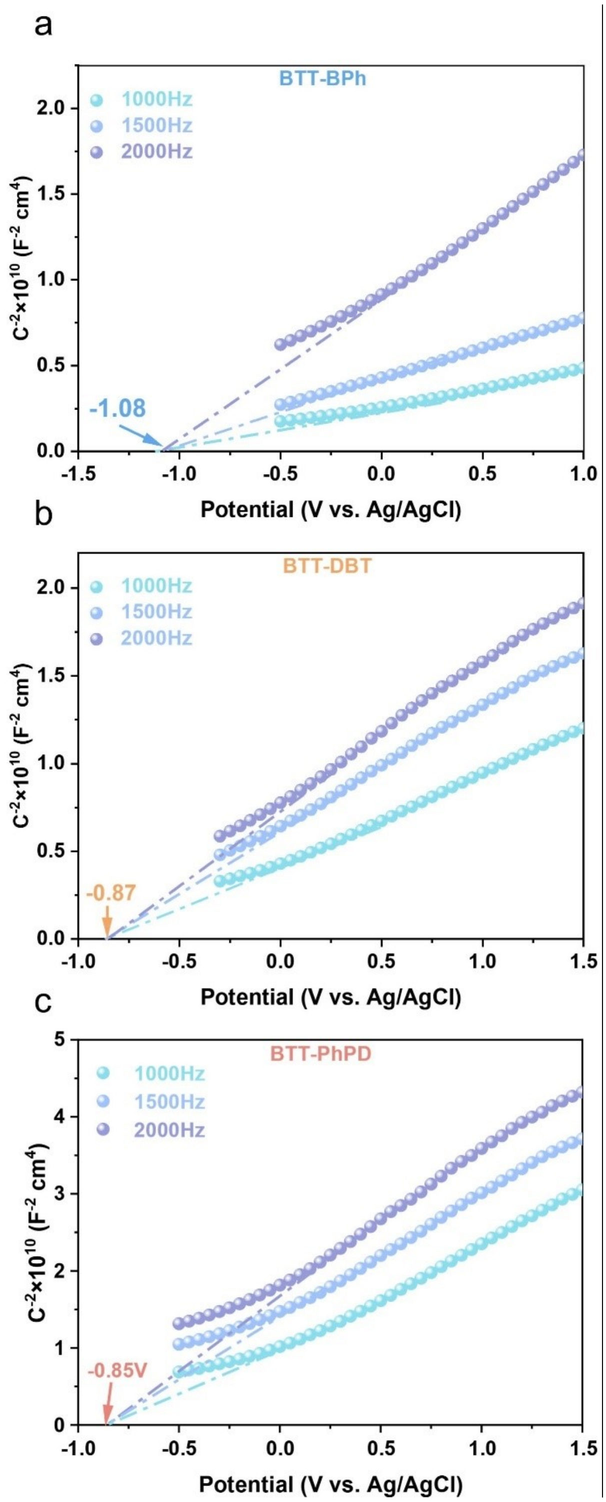

Fig. S8. Mott-Schottky plots of the COFs. the E_{CB} also can be determined by the equation: E_{CB} (vs. NHE pH=7) = E_{fb} (vs. Ag/AgCl) + 0.197.

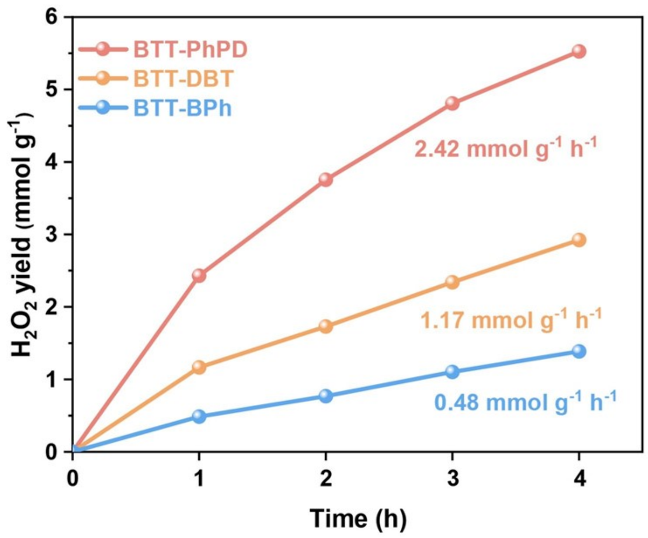

Fig. S9. H_2O_2 yield of the COFs in pure water under O_2 atmosphere.

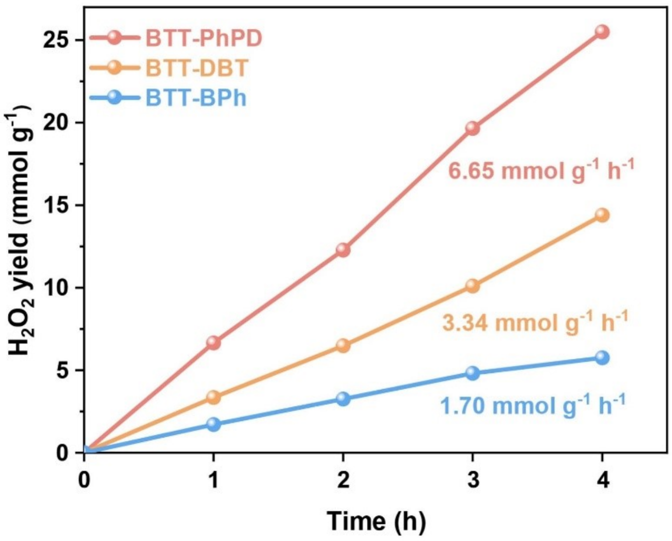

Fig. S10. H_2O_2 yield of the COFs in Benzyl alcohol solution (Benzyl alcohol: water= 1:9) under air atmosphere.

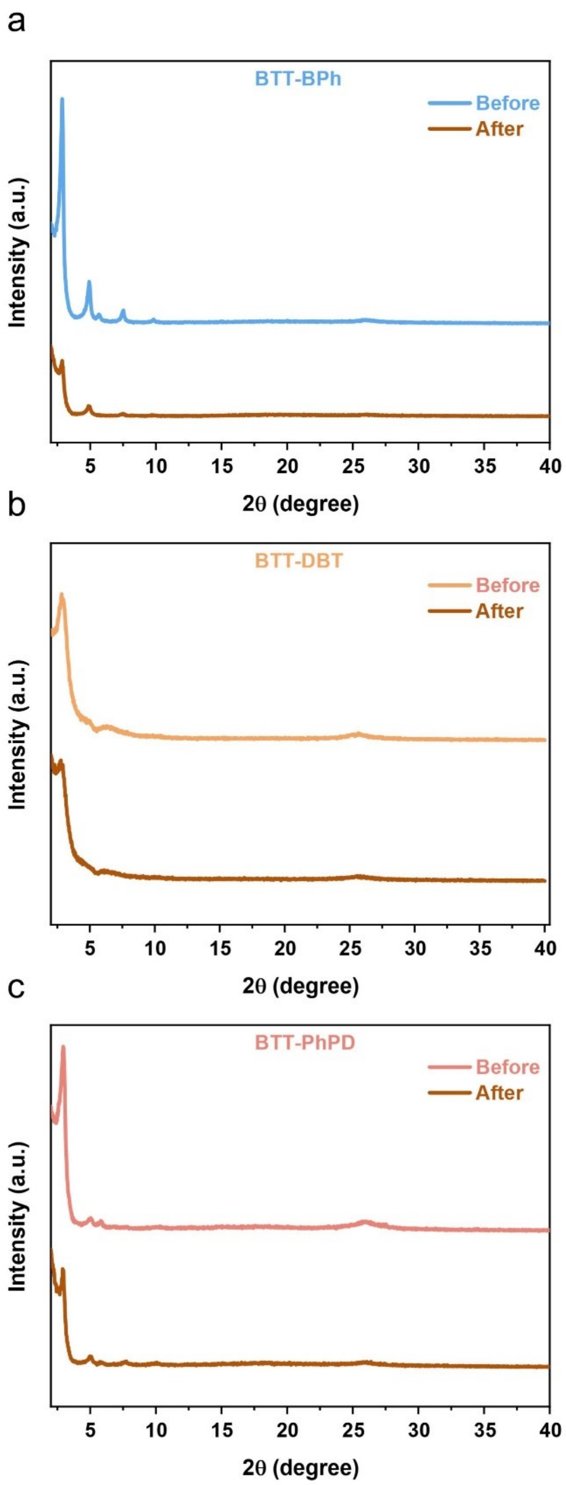

Fig. S11. PXRD patterns of the COFs before and after the photocatalytic tests.

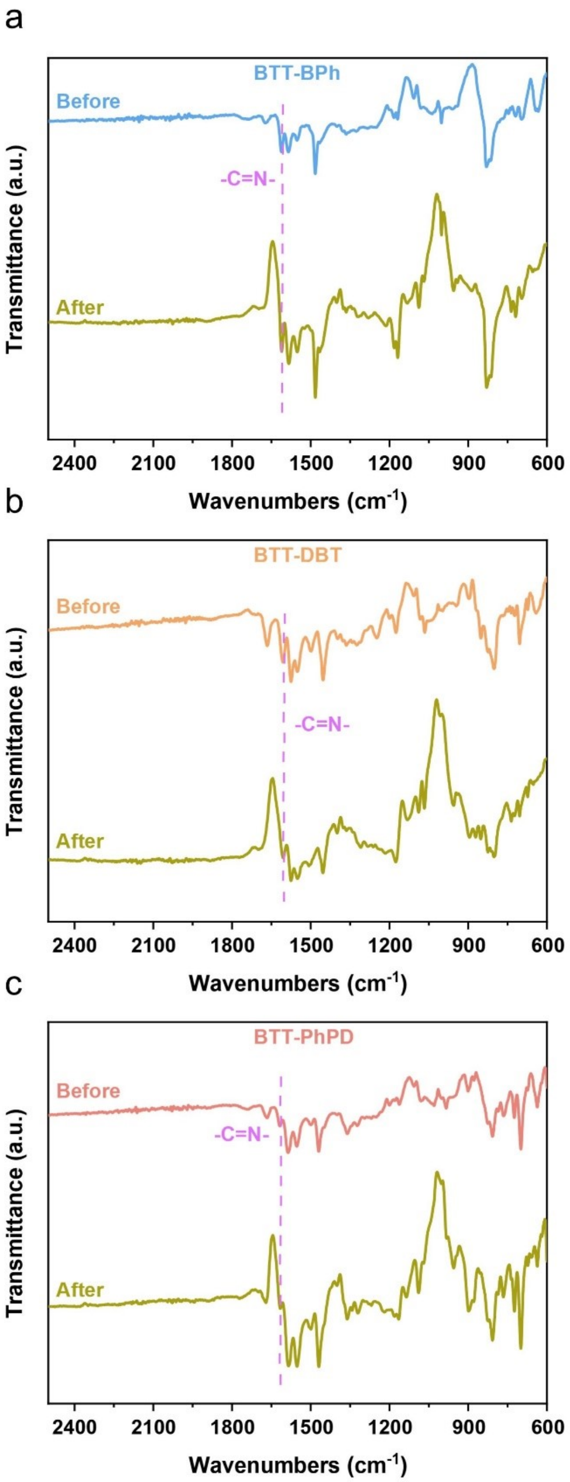

Fig. S12. FT-IR spectra of the COFs before and after the photocatalytic tests.

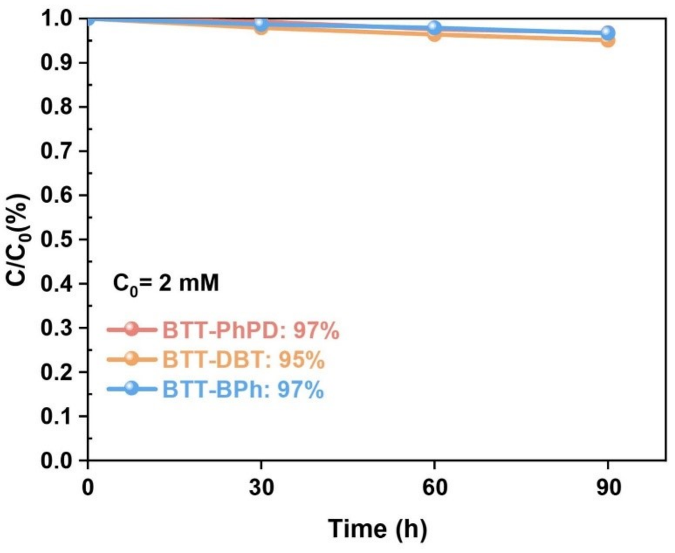

Fig. S13. Decomposition of H_2O_2 of the COFs.

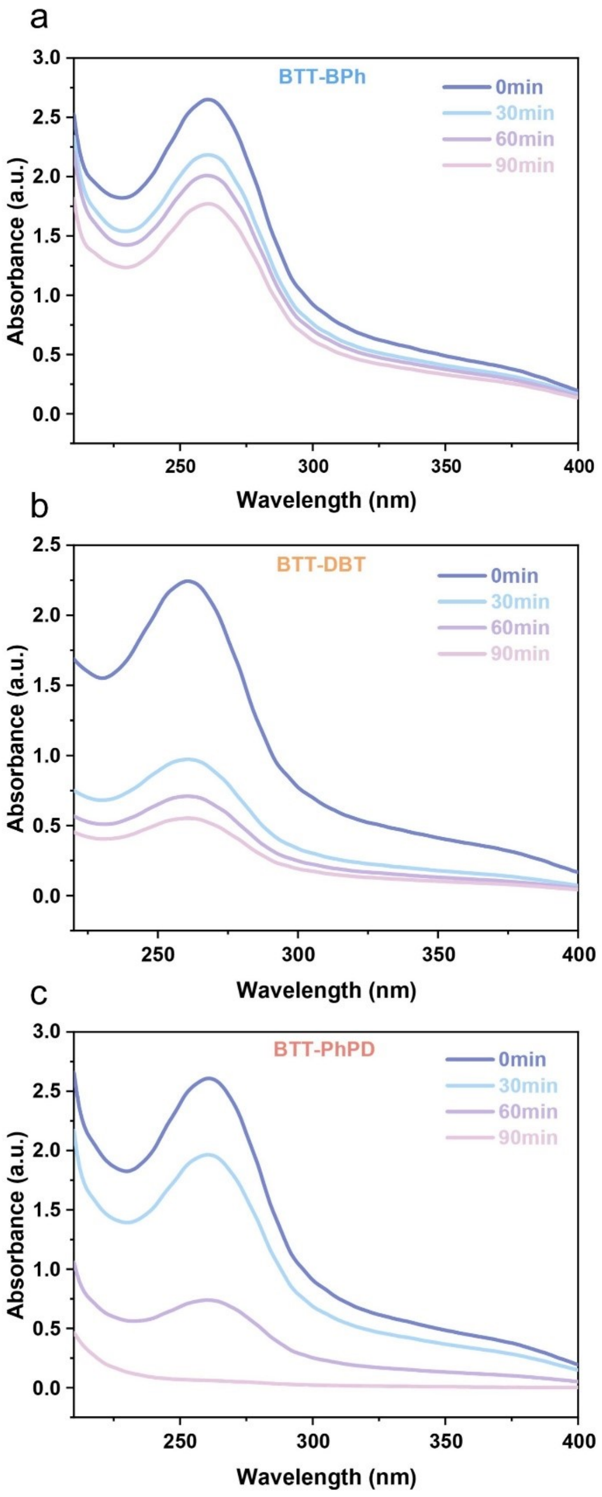

Fig. S14. Time-dependent absorption peak of NBT of the COFs.

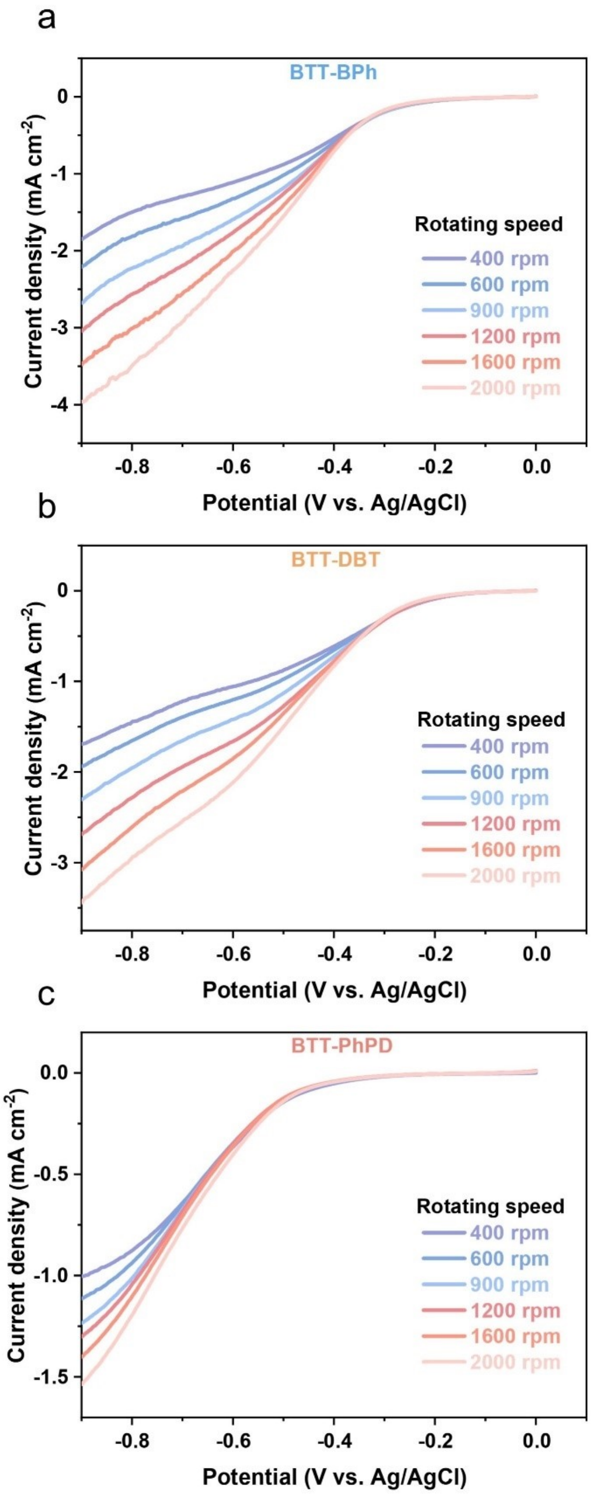

Fig. S15. LSV curves of the COFs measured on RDE at different rotating speeds.

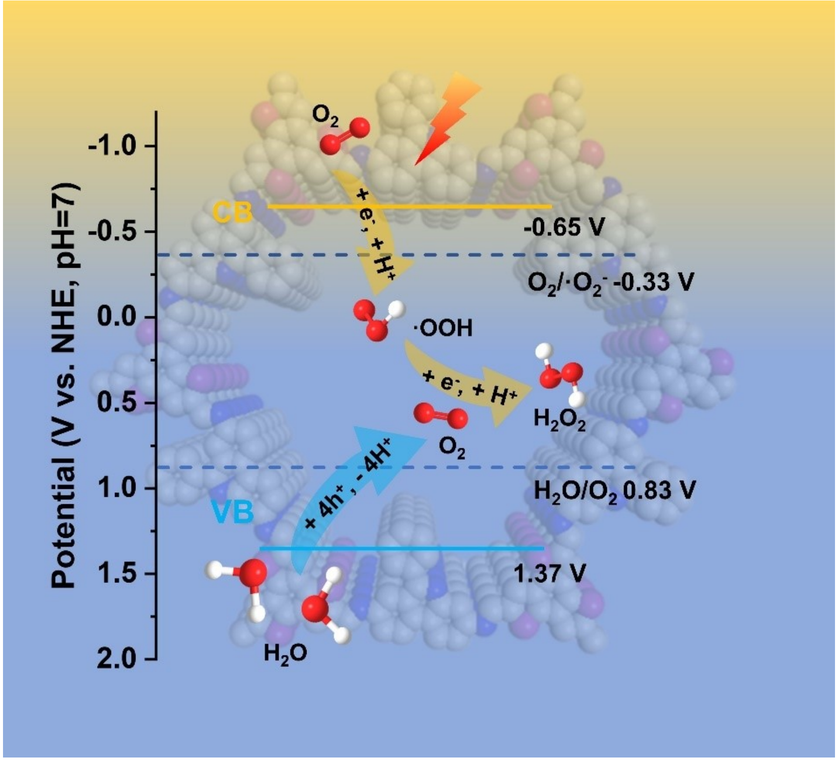

Fig. S16. Proposed mechanism of BTT-PhPD-based photocatalytic processes for the H_2O_2 production.

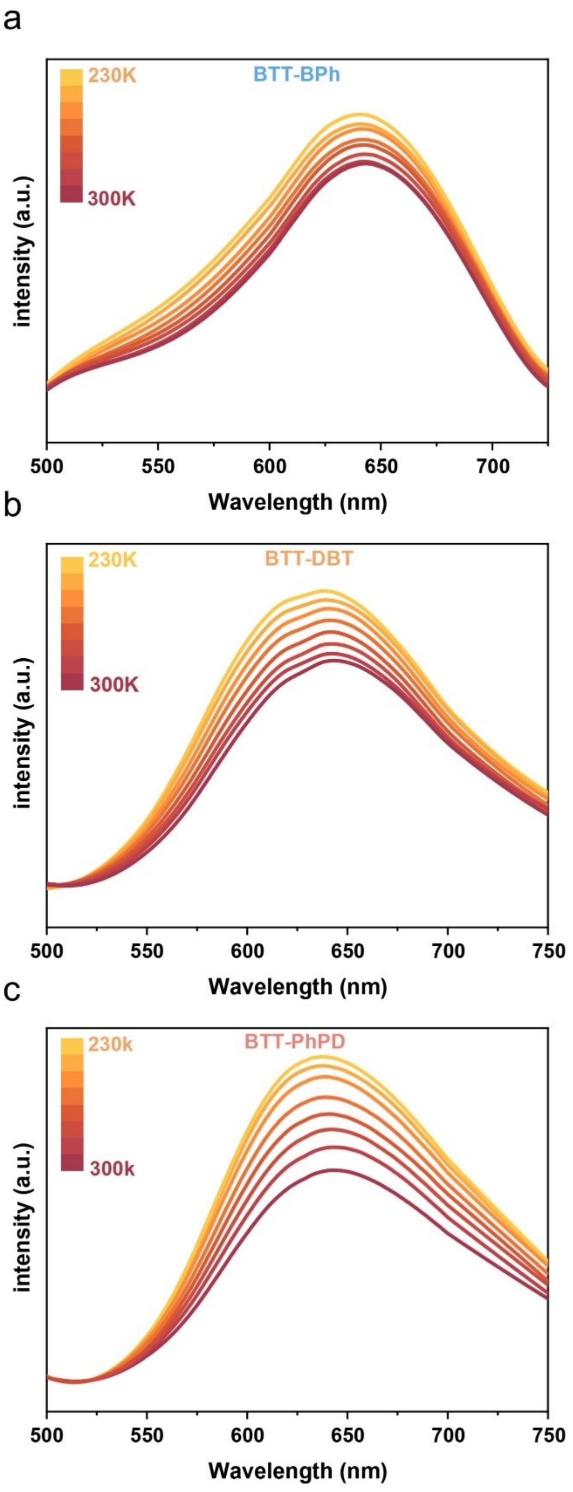

Fig. S17. Temperature-dependent PL spectra of the COFs.

.

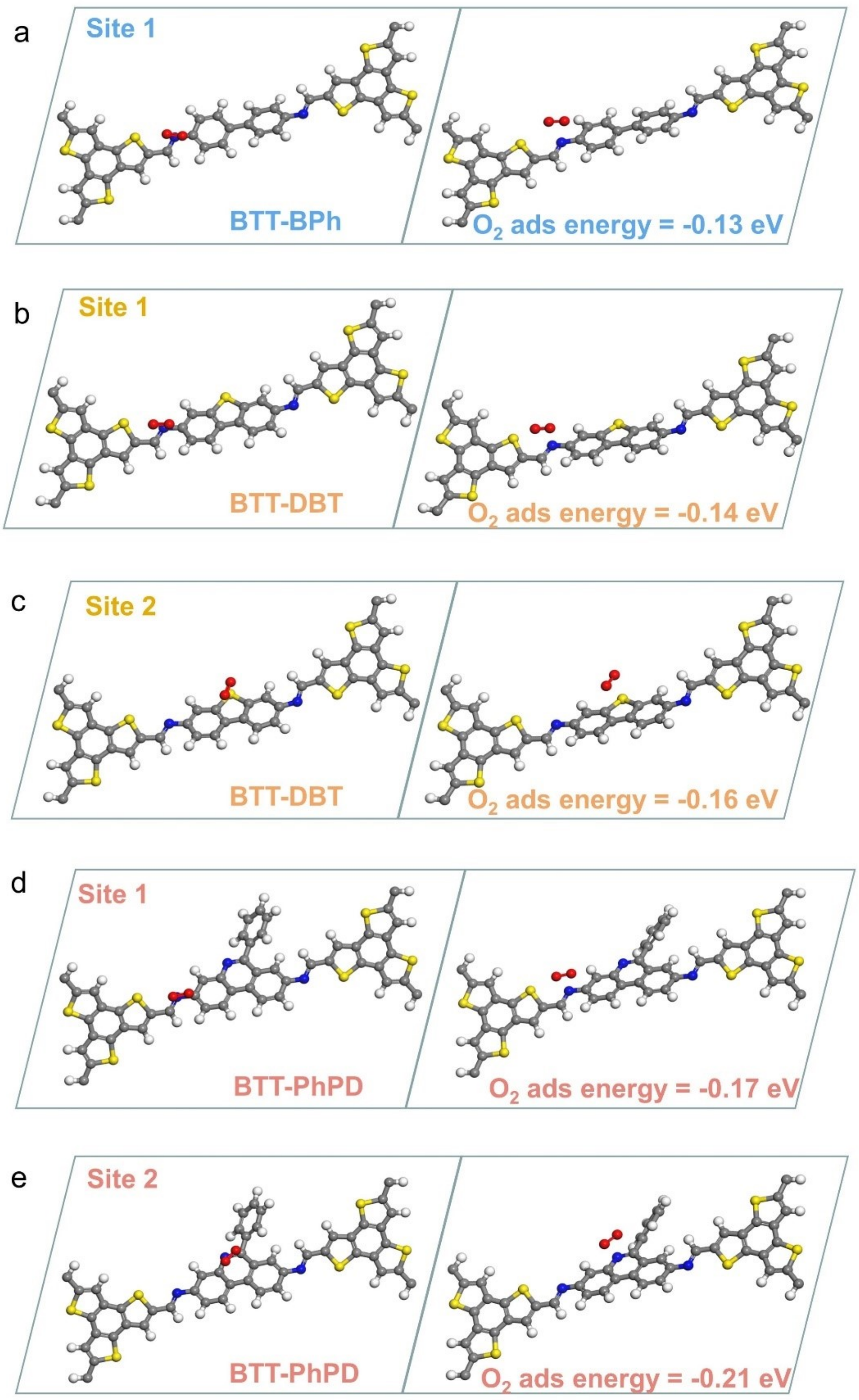

Fig. S18. Oxygen adsorption energy on different sites of the COFs.

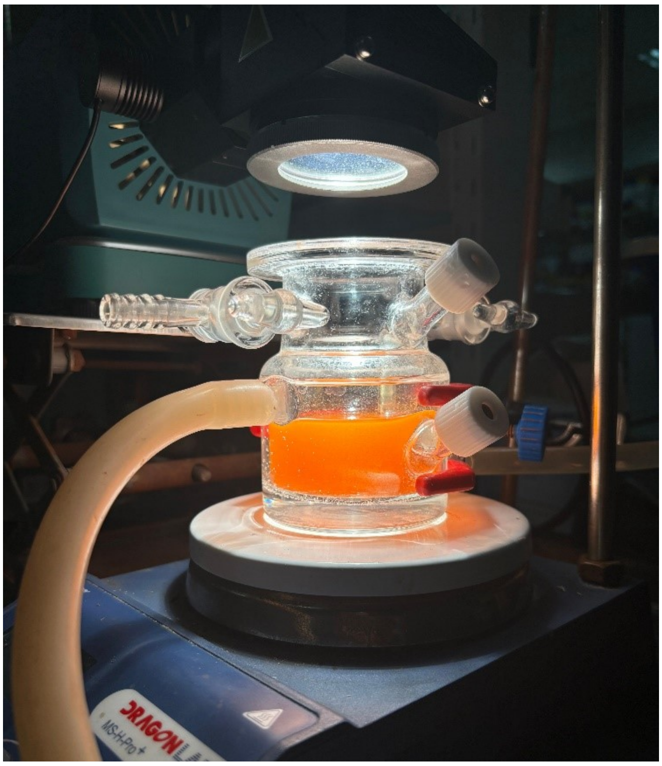

Fig. S19. The classic test system for the photocatalytic H_2O_2 production.

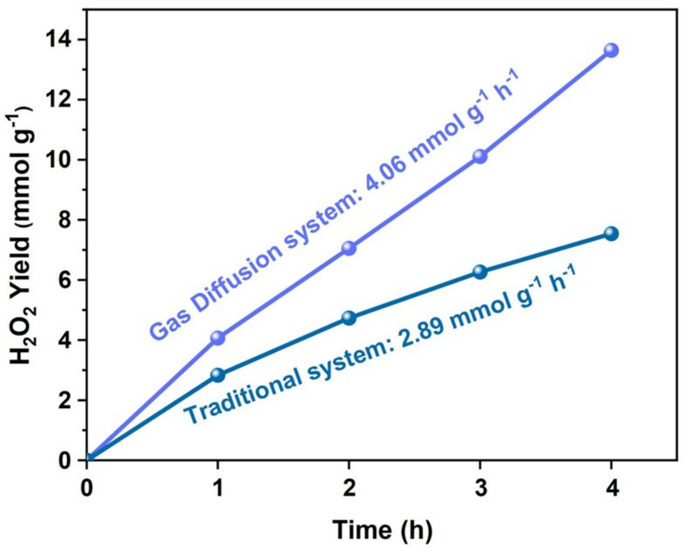

Fig. S20. The production of H_2O_2 within 4 hours in traditional system (1mg COF in 50 ml pure water) and gas diffusion flow System with 1 mg BTT-PhPD.

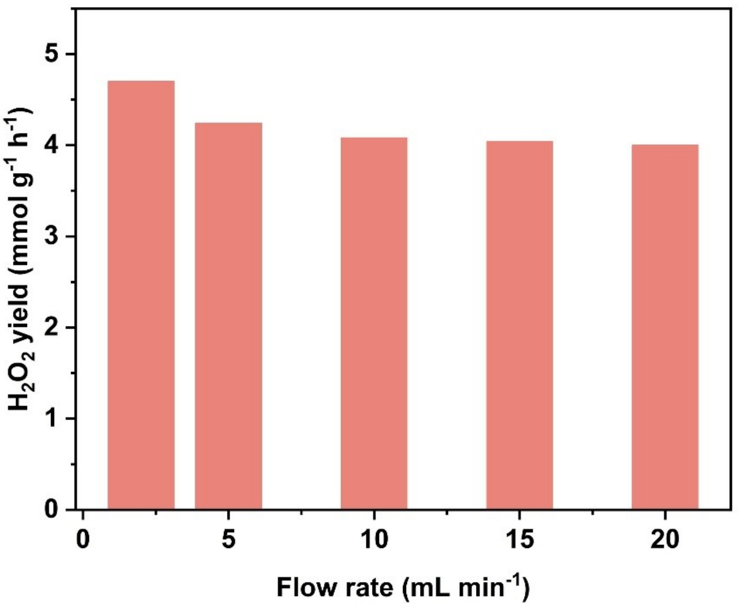

Fig. S21. Photocatalytic H_2O_2 yield at different water flow rates in the gas diffusion system.

Section 4. Tables

Table S1. Fractional atomic coordinates for BTT-BPh: space group P 6/m; *a*=*b*=35.8 Å, *c*=3.5 Å; *α*=*β*=90°; *γ*=120°.

**Atom**	**x (Å)**	**y (Å)**	**z (Å)**
C1	1.63736	-0.71031	0
C2	1.68096	-0.69639	0
C3	1.63365	-0.61263	0
C4	1.5894	-0.63897	0
S5	1.57012	-0.69339	0
C6	1.56142	-0.62079	0
N7	1.57935	-0.84145	0
C8	1.48858	-0.55713	0
C9	1.51054	-0.57985	0
C10	1.55567	-0.55738	0
C11	1.57867	-0.51251	0
C12	1.5567	-0.48979	0
C13	1.51154	-0.51197	0

Table S2. Fractional atomic coordinates for BTT-DBT: space group PM; *a*=36.02, *b*=35.99 Å, *c*=3.51 Å； *α*=*β*=90°； *γ*=120°.

**Atom**	**x (Å)**	**y (Å)**	**z (Å)**
N1	1.98046	-2.43869	0
C2	2.6613	-2.29855	0
C3	2.61729	-2.32546	0
S4	2.60083	-2.37916	0
C5	2.65392	-2.36506	0
C6	2.68241	-2.32136	0
C7	2.66885	-2.39416	0
C8	2.742	-2.33394	0
C9	2.72667	-2.305	0
C10	2.67957	-2.25403	0
C11	2.65227	-2.23677	0
C12	2.58978	-2.30859	0
C13	2.75649	-2.5407	0
C14	2.78297	-2.55832	0
C15	2.82766	-2.53172	0
C16	2.84552	-2.48749	0
C17	2.81883	-2.46936	0
C18	2.77463	-2.49598	0
S19	2.83509	-2.41593	0
C20	2.78183	-2.43007	0
C21	2.75292	-2.47377	0
S22	2.8641	-2.54866	0
C23	2.90375	-2.49572	0

C24	2.88963	-2.46615	0
S25	2.70306	-2.57762	0
C26	2.71693	-2.61689	0
C27	2.76059	-2.6023	0
C28	2.9497	-2.48473	0
C29	2.68426	-2.66253	0
C30	2.76844	-2.39769	0
N31	2.72818	-2.4088	0
C32	2.71322	-2.37864	0
C33	2.15776	-2.37047	0
C34	2.17503	-2.32644	0
S35	2.1378	-2.30989	0
C36	2.09862	-2.36303	0
C37	2.1139	-2.39147	0
C38	2.05446	-2.37811	0
C39	2.04196	-2.45115	0
C40	2.08615	-2.43573	0
C41	2.18378	-2.38899	0
C42	2.22832	-2.36192	0
C43	2.21949	-2.29909	0
C44	2.43421	-2.17288	0
C45	2.42743	-2.21441	0
C46	2.46235	-2.22159	0
C47	2.50371	-2.18689	0
C48	2.51083	-2.14489	0

C49	2.47631	-2.13791	0
S50	2.55965	-2.09935	0
C51	2.53329	-2.07067	0
C52	2.48879	-2.09504	0
S53	2.45981	-2.27029	0
C54	2.51497	-2.24316	0
C55	2.53435	-2.19882	0
S56	2.38823	-2.17015	0
C57	2.36	-2.22533	0
C58	2.38478	-2.24495	0
C59	2.53952	-2.26567	0
C60	2.31292	-2.2495	0
C61	2.55857	-2.02269	0
N62	2.58109	-2.24431	0
N63	2.29226	-2.29108	0
C64	2.60749	-2.26366	0
C65	2.24648	-2.3171	0
C66	2.02578	-2.42263	0
C67	2.58408	-2.86903	0
C68	2.61109	-2.88599	0
S69	2.66472	-2.84889	0
C70	2.65062	-2.80994	0
C71	2.60694	-2.82505	0
C72	2.67983	-2.766	0
C73	2.66454	-2.73699	0

C74	2.61989	-2.75268	0
C75	2.59076	-2.79696	0
C76	2.53954	-2.89554	0
C77	2.52247	-2.94018	0
C78	2.59445	-2.93024	0
N79	2.69497	-2.69197	0
N80	2.53155	-3.00332	0
C81	2.54969	-2.95772	0

Table S3. Fractional atomic coordinates for BTT-PhPD: space group PM; *a*=*b*=35.01 Å, *c*=3.50 Å; *α*=*β*=90°; *γ*=120°.

**Atom**	**x (Å)**	**y (Å)**	**z (Å)**
C1	1.28242	-0.25882	0
C2	1.30845	-0.21449	0
C3	1.28972	-0.18782	0
C4	1.24401	-0.20724	0
C5	1.21764	-0.2527	0
C6	1.31755	-0.14274	0
C7	1.36332	-0.12577	0
C8	1.39042	-0.08115	0
C9	1.32922	-0.06919	0
C10	1.30096	-0.11412	0
C11	1.37408	-0.05318	0
C12	1.76024	-0.7831	0
C13	1.80516	-0.76641	0
C14	1.83385	-0.72114	0
C15	1.81696	-0.69231	0
C16	1.77235	-0.70986	0
C17	1.87893	-0.70387	0
C18	1.89828	-0.73018	0
C19	1.94373	-0.71106	0
C20	1.95	-0.64008	0
C21	1.90567	-0.65841	0
C22	1.96974	-0.66571	0

C23	1.53397	-0.78635	0
C24	1.49025	-0.81745	0
C25	1.47747	-0.86146	0
C26	1.50852	-0.87408	0
C27	1.55255	-0.84279	0
C28	1.56523	-0.79907	0
C29	1.58673	-0.8503	0
C30	1.62476	-0.8108	0
S31	1.6195	-0.76507	0
C32	1.54151	-0.74462	0
C33	1.50201	-0.74617	0
S34	1.45627	-0.79725	0
C35	1.43571	-0.89568	0
C36	1.43716	-0.93366	0
S37	1.4882	-0.92834	0
C38	1.66737	-0.80687	0
C39	1.49806	-0.7075	0
C40	1.39842	-0.9763	0
N41	1.69964	-0.7677	0
C42	1.74431	-0.75423	0
N43	1.40531	-1.00852	0
C44	1.14355	-0.51297	0
C45	1.15867	-0.54207	0
C46	1.20337	-0.5261	0
C47	1.2325	-0.48182	0

C48	1.2166	-0.45309	0
C49	1.17234	-0.46826	0
C50	1.24071	-0.40913	0
C51	1.21331	-0.39233	0
S52	1.1585	-0.42872	0
C53	1.0996	-0.53286	0
C54	1.08274	-0.57713	0
S55	1.11908	-0.59553	0
C56	1.22326	-0.55012	0
C57	1.26753	-0.52259	0
S58	1.28595	-0.46783	0
C59	1.23253	-0.34595	0
C60	1.03634	-0.60434	0
C61	1.2948	-0.54162	0
N62	1.2114	-0.32506	0
C63	1.23701	-0.27863	0
C64	1.33198	-0.59126	0
C65	1.34928	-0.61828	0
C66	1.39515	-0.59907	0
C67	1.4216	-0.55334	0
C68	1.40288	-0.52677	0
C69	1.35768	-0.54595	0
N70	1.33688	-0.52037	0
C71	1.41318	-0.62617	0
C72	1.38476	-0.6716	0

C73	1.40113	-0.69943	0
C74	1.44556	-0.68318	0
C75	1.47473	-0.63822	0
C76	1.45841	-0.60965	0
N77	1.4589	-0.71445	0
N78	1.01539	-0.6464	0
N79	1.88878	-0.63176	0
C80	1.84544	-0.64666	0
N81	1.34113	-0.68885	0
C82	1.32195	-0.66413	0
C83	1.38052	-0.15417	0
N84	1.35205	-0.19752	0
C85	1.83141	-0.6133	0
C86	1.2732	-0.6876	0
C87	1.42797	-0.14004	0
C88	1.8634	-0.56894	0
C89	1.85192	-0.53661	0
C90	1.80811	-0.54815	0
C91	1.77586	-0.592	0
C92	1.78749	-0.62421	0
C93	1.24916	-0.66595	0
C94	1.2037	-0.68911	0
C95	1.18142	-0.73453	0
C96	1.20465	-0.75668	0
C97	1.25013	-0.73336	0

C98	1.44036	-0.17196	0
C99	1.48422	-0.16038	0
C100	1.51651	-0.11655	0
C101	1.5049	-0.08437	0
C102	1.46101	-0.09609	0

Table S4. Comparison of the photocatalytic H_2O_2 production performance of BBT-PhPD with other reported COF-based photocatalysts under similar test condition.

Sample Name	H_2O_2 evolution rate (mmol g^{-1} h^{-1})	Solvent mixture	Reference
BBT-PhPD	2.04	Water, Air	This work
	2.42	Water, O_2	
	6.65	Water:BA= 9:1, Air	
BTT-PhPD-gas diffusion system	4.08	Water, Air	
COF-TPT-Azo	1.498	Water, PH=11, Air	7
TTA-Azo-COF	2.516	Water, O_2	8
COF-JLU90	6.432	Water, O_2	9
CTF-BTT	6.230	Water, O_2	10
iTPPy-COF	7.955	Water, O_2	11
EBBT-COF	5.686	Water, O_2	12
g-COF-DMDP-1	3.82	Water, O_2	13
PD-COF2	6.103	Water, air	14
TMT-TT	1.952	Water, O_2	15
COF-BTT-TATP	0.62	Water, O_2	16
4PE−N−S COF	1.574	Water, O_2	17
HEP−TAPT−COF	1.750	Water, air	18
COF-JLU-52	4.200	Water, O_2	19
β-TT-TDAN COF	3.424	Water, O_2	20
TBA-COF	8.878	Water, Air	21
TPB-COF-OH	6.608	Water, Air	22
PD-COF2	6.103	Water, Air	23
FS-COFs	3.904	Water, Air	24

References

1. W. Zhao, P. Y. Yan, B. Y. Li, M. Bahri, L. J. Liu, X. Zhou, R. Clowes, N. D. Browning, Y. Wu, J. W. Ward, A. Cooper, *J. Am. Chem. Soc.* **2022**, 144, 9902.
2. E. Lebègue, *Transition Met. Chem.* **2023**, 48, 433.
3. L. Hao, R. C. Shen, C. Huang, Z. Z. Liang, N. Li, P. Zhang, X. Z. Li, C. C. Qin, X. Li, *Appl. Catal. B-Environ.* **2023**, 330, 122581.
4. H. Wei, J. Ning, X. Cao, X. Li, L. Hao, *J. Am. Chem. Soc.* **2018**, 140, 11618.
5. Q. Tang, Y.-Y. Gu, J. Ning, Y. Yan, L. Shi, M. Zhou, H. Wei, X. Ren, X. Li, J. Wang, C. Tang, L. Hao, J. Ye, *Chem. Eng. J.* **2023**, 470, 144106.
6. D. Bruns, H. Miura, K. P. C. Vollhardt, A. Stanger, *Org. Lett.* **2003**, 5, 549.
7. H. H. Sun, Z. B. Zhou, Y. B. Fu, Q. Y. Qi, Z. X. Wang, S. Q. Xu, X. Zhao, *Angew. Chem. Int. Ed.* **2024**, *63*, e202409250.
8. J.-Z. Xiao, Z.-H. Zhao, N.-N. Zhang, H.-T. Che, X. Qiao, G.-Y. Zhang, X. Chu, Y. Wang, H. Dong, F.-M. Zhang, *Chin. J. Catal.* **2025**, *69*, 219-229.
9. Z. Zhang, Y. Hou, S. Zhu, L. Yang, Y. Wang, H. Yue, H. Xia, G. Wu, S. W. Yang, X. Liu, *Angew. Chem. Int. Ed.* **2025**, e202505286. http://doi.org/10.1002/anie.202505286.
10. R. Sun, X. Yang, X. Hu, Y. Guo, Y. Zhang, C. Shu, X. Yang, H. Gao, X. Wang, I. Hussain, B. Tan, *Angew. Chem. Int. Ed.* **2025**, *64*, e202416350.
11. J. Zhang, F. Xue, Z. Wang, *Angew. Chem. Int. Ed.* **2025**, *64*, e202425617.
12. B. Li, J. Chen, K. Wang, D. Qi, T. Wang, J. Jiang, *Adv. Energy Mater.* **2025**, *15*, 2404497.
13. X. Chi, Z. Zhang, M. Li, Y. Jiao, X. Li, F. Meng, B. Xue, D. Wu, F. Zhang, *Angew. Chem. Int. Ed.* **2025**, *64*, e202418895.
14. J. Y. Yue, J. X. Luo, Z. X. Pan, Q. Xu, P. Yang, B. Tang, *Angew. Chem. Int. Ed.* **2025**, *64*, e202417115.
15. M. Deng, L. Wang, Z. Wen, J. Chakraborty, J. Sun, G. Wang, P. Van Der Voort, *Green Chem.* **2024**, 26, 3239.
16. M. J. Liu, P. P. He, H. T. Gong, Z. H. Zhao, Y. M. Li, K. Zhou, Y. M. Lin, J. Li, Z. B.

Bao, Q. W. Yang, Y. W. Yang, Q. L. Ren, Z. G. Zhang, *Chem. Eng. J.* **2024**, 482, 148922.

17. M. Deng, J. Sun, A. Laemont, C. Liu, L. Wang, L. Bourda, J. Chakraborty, K. Van Hecke, R. Morent, N. De Geyter, K. Leus, H. Chen, P. Van Der Voort, *Green Chem.* **2023**, 25, 3069.
18. D. Chen, W. Chen, Y. Wu, L. Wang, X. Wu, H. Xu, L. Chen, *Angew. Chem. Int. Ed.* **2023**, 62 , e202217479.
19. X. Liu, Z. Zhang, Q. Zhang, Y. Hou, J. Li, S. Zhu, H. Xia, H. Yue, *Angew. Chem. Int. Ed.* **2024**, 63, e202411546.
20. W. Zhang, M. Sun, J. Cheng, X. Wu, H. Xu, *Adv. Mater.* **2025**, *37*, e2500913.
21. J. Y. Yue, Z. X. Pan, R. Z. Zhang, Q. Xu, P. Yang, B. Tang, *Adv. Funct. Mater.* **2025**, 35, 202421514.
22. S. Feng, H. Cheng, F. Chen, X. Liu, Z. Wang, H. Xu, J. Hua, *ACS Catal.* **2024**, 14, 7736.
23. J. Y. Yue, J. X. Luo, Z. X. Pan, Q. Xu, P. Yang, B. Tang, *Angew. Chem. Int. Ed.* **2025**, 64, e202417115.
24. Y. Luo, B. Zhang, C. Liu, D. Xia, X. Ou, Y. Cai, Y. Zhou, J. Jiang, B. Han, *Angew. Chem. Int. Ed.* **2023**, 62, e202305355.
